# Antiplasmodial and Antileishmanial Activities of a New Limonoid and Other Constituents from the Stem Bark of *Khaya senegalensis*

**DOI:** 10.3390/molecules28207227

**Published:** 2023-10-23

**Authors:** Gabrielle Ange Amang à Ngnoung, Yves Oscar Nganso Ditchou, Peron Bosco Leutcha, Darline Dize, Simplice Joël Ndendoung Tatsimo, Lauve Rachel Yamthe Tchokouaha, Theodora Kopa Kowa, Babalwa Tembeni, Hamadou Mamoudou, Madan Poka, Patrick Hulisani Demana, Xavier Siwe Noundou, Fabrice Fekam Boyom, Alain Meli Lannang

**Affiliations:** 1 Department of Chemistry, Faculty of Science, University of Maroua, Maroua P.O. Box 814, Cameroon; gabrielleangeamang@gmail.com (G.A.A.à.N.); peron.leutcha@gmail.com (P.B.L.); 2 Natural Product and Environmental Chemistry Group (NAPEC), Department of Chemistry, Higher Teachers’ Training College, University of Maroua, Maroua P.O. Box 55, Cameroon; jtatsimo@yahoo.com (S.J.N.T.); alainmeli@yahoo.com (A.M.L.); 3 Antimicrobial and Biocontrol Agents Unit (AmBcAU), Laboratory for Phytobiochemistry and Medicinal Plants Studies, Department of Biochemistry, Faculty of Science, University of Yaoundé I, Yaoundé P.O. Box 812, Cameroon; darline.dize@yahoo.fr (D.D.); fabrice.boyom@fulbrightmail.org (F.F.B.); 4 Laboratory of Pharmacology, Centre for Research on Medicinal Plants and Traditional Medicine, Institute of Medical Research and Medicinal Plants Studies, Yaoundé P.O. Box 13033, Cameroon; yamthe_lauve@yahoo.fr; 5 Laboratory of Phytochemistry, Centre for Research on Medicinal Plants and Traditional Medicine, Institute of Medical Research and Medicinal Plants Studies, Yaoundé P.O. Box 13033, Cameroon; kothera81@yahoo.fr; 6 Department of Pharmaceutical Sciences, School of Pharmacy, Sefako Makgatho Health Sciences University, Pretoria 0204, South Africa; btembeni2@gmail.com (B.T.); madan.poka@smu.ac.za (M.P.); patrick.demana@smu.ac.za (P.H.D.); 7 Department of Biological Sciences, Faculty of Science, University of Maroua, Maroua P.O. Box 814, Cameroon; mamoudhmd@gmail.com; 8 Advanced Research and Health Innovation Hub (ARHIH), Yaoundé P.O. Box 20133, Cameroon; 9 Department of Chemical Engineering, School of Chemical Engineering and Mineral Industries, University of Ngaoundere, Ngaoundere P.O. Box 454, Cameroon

**Keywords:** Meliaceae, *Khaya senegalensis*, limonoids, antileishmanial activity, antiplasmodial activity

## Abstract

*Plasmodium falciparum* and *Leishmania* sp. resistance to antiparasitic drugs has become a major concern in malaria and leishmaniasis control. These diseases are public health problems with significant socioeconomic impacts, and mostly affect disadvantaged populations living in remote tropical areas. This challenge emphasizes the need to search for new chemical scaffolds that preferably possess novel modes of action to contribute to antimalarial and antileishmanial research programs. This study aimed to investigate the antimalarial and antileishmanial properties of a methanol extract (**KS**-**MeOH**) of the stem bark of the Cameroonian medicinal plant *Khaya senegalensis* and its isolated compounds. The purification of **KS**-**MeOH** led to the isolation of a new ordered limonoid derivative, 21*β*-hydroxybourjotinolone A (**1a**), together with 15 known compounds (**1bc**–**14**) using a repeated column chromatography. Compound **1a** was obtained in an epimeric mixture of 21*α*-melianodiol (**1b**) and 21*β*-melianodiol (**1c**). Structural characterization of the isolated compounds was achieved with HRMS, and 1D- and 2D-NMR analyses. The extracts and compounds were screened using pre-established in vitro methods against synchronized ring stage cultures of the multidrug-resistant Dd2 and chloroquine-sensitive/sulfadoxine-resistant 3D7 strains of *Plasmodium falciparum* and the promastigote form of *Leishmania donovani* (1S(MHOM/SD/62/1S). In addition, the samples were tested for cytotoxicity against RAW 264.7 macrophages. Positive controls consisted of artemisinin and chloroquine for *P. falciparum*, amphotericin B for *L. donovani*, and podophyllotoxin for cytotoxicity against RAW 264.7 cells. The extract and fractions exhibited moderate to potent antileishmanial activity with 50% inhibitory concentrations (IC_50_) ranging from 5.99 ± 0.77 to 2.68 ± 0.42 μg/mL, while compounds displayed IC_50_ values ranging from 81.73 ± 0.12 to 6.43 ± 0.06 μg/mL. They were weakly active against the chloroquine-sensitive/sulfadoxine-resistant *Pf*3D7 strain but highly potent toward the multidrug-resistant *Pf*Dd2 (extracts, IC_50_ 2.50 ± 0.12 to 4.78 ± 0.36 μg/mL; compounds IC_50_ 2.93 ± 0.02 to 50.97 ± 0.37 μg/mL) with selectivity indices greater than 10 (SI_Dd2_ > 10) for the extract and fractions and most of the derived compounds. Of note, the limonoid mixture [21*β*-hydroxylbourjotinolone A (**1a**) + 21*α*-melianodiol (**1b**) + 21*β*-melianodiol (**1c**)] exhibited moderate activity against *P. falciparum* and *L. donovani.* This novel antiplasmodial and antileishmanial chemical scaffold qualifies as a promising starting point for further medicinal chemistry-driven development of a dually active agent against two major infectious diseases affecting humans in Africa.

## 1. Introduction

Secondary metabolites play an important role in the identification of plants through chemotaxonomy, and exhibit a panel of bioactivities. Secondary metabolites are reported from soil, animals, fungi, bacteria, and green plants, and they are grouped into several classes and subclasses, such as terpenoids, flavonoids, and polyketides. The presence, type, and abundance of these compounds are usually related to the origin, species, and environment where the studied samples were collected. The search for secondary metabolites began when scientists examined the factors responsible for the sweetness or bitterness of fruit [1,2]. Limonoids are a subclass of well-known triterpenoids in the plant kingdom, and are among the secondary metabolites responsible for the bitter taste of fruit. The occurrence of limonoids in the plant kingdom is limited to plants of the Rutales order, and they are most abundant in the Rutaceae and Meliaceae families [1,3]. They are less abundant in the Simaroubaceae and Cneoraceae families [2]. The structural modification of Rutaceae limonoids is usually restricted to the modification of A and B rings, while Meliaceae limonoids are the most complex limonoids of the Rutale order with a high oxidation degree [2,3]. Limonoids are usually divided into two main groups, of which the first group contains a moiety of a well-known triterpenoid called an ordered limonoid, while the second group is called the degraded (modified) triterpenoid without a fixed skeleton. Both groups of limonoids are highly oxygenated with a skeleton of 4,4,8-trimethyl-17-furanylsteroid or a protolimonoid as a prototypical precursor [2,4]. Almost all naturally reported limonoids contain either a furan or a pyran ring linked to the D-ring at C-17, and the preferable oxygenated carbons are C23, C21, C-17, C-16, C-7, C-4, C-3, and C1 [2,5,6,7,8]. Limonoids exhibit a panel of biological activities, such as insect antifeedant, and insecticidal, antimalarial, antimicrobial, antiviral, and anticancer activities [2,3,9].

*Khaya senegalensis* (Desr.) A. Juss. (Meliaceae) is a plant species known as Caïlcédrat (French). *K. senegalensis* is a tree of approximately 25 m in height whose leaves, fruit, and stems are largely used by many African tribes in Cameroon and Nigeria for the treatment of malaria, headache, and yellow fever [9,10,11,12]. Previous chemical and biological studies of different parts of *K. senegalensis* have revealed the occurrence of flavonoids, alkaloids, triterpenoids, steroids, and limonoids with cytotoxic, antimicrobial, hepatoprotective, antiviral, and antioxidant activities [2,9,11,12,13]. Limonoids are reported to be the most abundant and chemophenetic taxon of *K. senegalensis* as well as the *Khaya* genus [3,13]. In our search for bioactive compounds from medicinal plants growing in Cameroon [11,14,15], we have undertaken a chemical investigation of the methanol extract of the stem bark of *K. senegalensis*. This study aimed to investigate the antimalarial and antileishmanial properties of the extract and fractions of the stem bark of the Cameroonian medicinal plant *Khaya senegalensis* as well as its isolated compounds. This investigation led to the isolation of 16 secondary metabolites (**1abc**–**14**). All of the isolated compounds were characterized by NMR and MS techniques, and compound 1a was characterized as an unprecedentedly ordered limonoid-type bourjotinolone. The chemophenetic aspect was also discussed. In addition, the methanolic crude extract, the EtOAc and *n*-hexane fractions and the isolated compounds (**1abc**–**14**) were subjected to antiplasmodial, antileishmanial, and cytotoxicity testing.

## 2. Results

**Figure 1:**
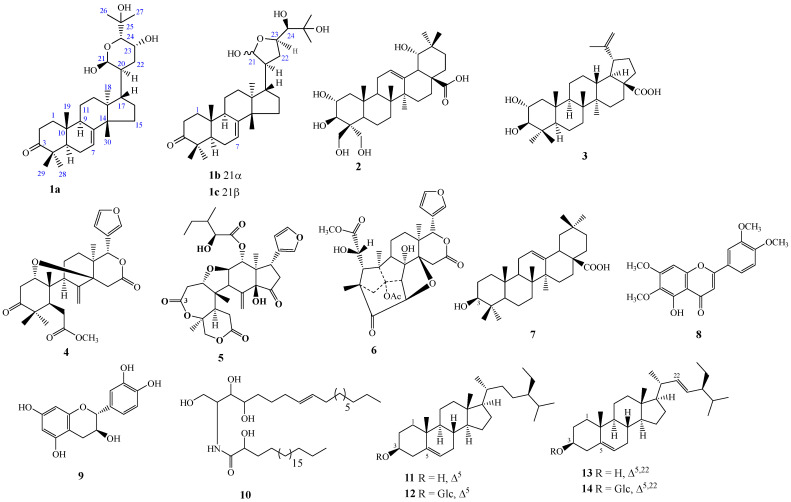
Compounds isolated from the stem bark of *K. senegalensis* **1abc**–**14**.

A mass of 275.0 g of the MeOH extract of *K. senegalensis* was partitioned into *n*-hexane (15.6 g) and EtOAc (160.8 g) fractions. Repeated column chromatography with Sephadex as well as silica gel as the stationary phase of the EtOAc (160.0 g) and *n*-hexane (15.0 g) extracts led to the isolation of 16 secondary metabolites, including nine triterpenoids (**1abc**–**7**), four steroids (**11**–**14**), two flavonoids (**8**–**9**) and one ceramide (**10**) (Figure 1).

### 2.1. Structure Elucidation of Compound **1**

Compound **1** was isolated as a shiny white powder whose molecular formula was established as C_30_H_48_O_5_; HR-ESI-MS (positive) *m*/*z* 511.3394 [M + Na]^+^; calcd. *m*/*z* 511.3399 (for C_30_H_48_O_5_Na^+^), indicating seven double bond equivalents (Figure S1). The UV spectrum (Figure S2) of compound **1** showed absorption maxima at 268 and 365 nm, while its IR spectrum (Figure S3) revealed a broad absorption band ranging from 3669 to 3200 cm^−1^ attributable to hydroxyl groups and other important absorption bands at 1697 and 1488 cm^−1^ assigned to carboxyl and olefinic functions, respectively.

The ^1^H-NMR spectrum of compound **1** exhibited the profile of a triterpenoid derivative. The combination of the proton and carbon-13 NMR spectra of compound **1** led to its identification as an inseparable mixture of isomers. The ^1^H-NMR data of compound **1** were identical to those of melianodiol (**1b**) and 21-*epi*-melianodiol (**1c**) as well as the bourjotinolone A skeleton, with few differences, such as the appearance of signals at *δ*_H_ 5.26, 4.10 and 3.60 [16,17,18,19]. In addition, the signals of three olefinic protons were observed at *δ*_H_ 5.35 (3H, brq, *J* = 3.1 Hz, H-7abc) as well as typical oxymethine protons at *δ*_H_ 5.26 (1H, brd, *J* = 2.8 Hz, H-21a), 5.21 (2H, brdd, *J* = 4.0, 1.8 Hz, H-21bc), 4.46 (1H, brddd, *J* = 9.3, 6.7, 1.8 Hz, H-23c), 4.34 (1H, brddd, *J* = 10.8, 5.0, 2.3 Hz, H-23b), 4.10 (1H, brt, *J* = 3.6 Hz, H-23a), 3.60 (1H, brd, *J* = 1.5 Hz, H-24a), 3.20 (1H, brd, *J* = 2.3 Hz, H-24b), and 3.14 (1H, brd, *J* = 1.8 Hz, H-24c), which were in agreement with the three characteristic signals integrating for 21 angular methyl groups at *δ*_H_ [0.89 (3H, s, Me-18c), 0.91 (3H, s, Me-18a), 0.92 (3H, s, Me-18b)], [1.03 (9H, s), Me-28abc], [1.05 (9H, s, Me-19abc)], [1.06 (9H, s, Me-30abc)], [1.13 (9H, s, Me-29abc)], [1.24 (3H, s, Me-26a), 1.21 (3H, s, Me-26b), 1.19 (3H, s, Me-26c)], and [1.27 (3H, s, Me-27a). In addition, 1.26 (3H, s, Me-27b) and 1.23 (3H, s, Me-27c)] were also observed on ^1^H-NMR (Table 1, Figure S4), confirming the presence of three triterpenoid moieties. The ^13^C-NMR (Table 1, Figure S6a,b) spectrum of compound **1** showed overlapping carbon signals from C1 to C16 for the three triterpenoids due to the similar chemical environment.

The HSQC spectrum (Figure S7a,b) revealed characteristic correlations of an olefinic carbon at *δ*_C_ 119.2 (C-7)/*δ*_H_ 5.35 (H-7) and furanic carbons at *δ*_C_ 103.2 (C-21α)/*δ*_H_ 5.21 (H-21*α*), 94.1 (C-21*β*)/*δ*_H_ 5.21 (H-21*β*), 77.0 (C-23*α*)/*δ*_H_ 4.34 (H-23*α*), and 78.3 (C-23*β*)/*δ*_H_ 4.46 (H-23*β*) assigned to the furan moieties of 21*α/β*-melianodiol. The existence of 21*α/β*-melianodiol might be due to mutarotation with the formation of an equilibrium mixture of C-21 epimers [17,18,20]. In addition, the characteristic signals of a pyranic ring at *δ*_C_ 94.1 (C-21)/*δ*_H_ 5.26 (H-21) and 73.6 (C-24)/*δ*_H_ 3.60 (H-24) were also noticed and attributable to the bourjotinolone derivative with little difference, such as the appearance of the signal at *δ*_C_ 67.1 (C-23)/*δ*_H_ 4.10 (H-23) [4,16]. Moreover, the oxymethine carbons observed at *δ*_C_ 78.5 (C-24*α*)/*δ*_H_ 3.20 (H-24*α*) and 78.5 (C-24*β*)/*δ*_H_ 3.14 (H-24*β*) were also in agreement with the limonoid-type bourjotinolone and melianodiol derivatives [4,17,18,19].

**Figure 2:**
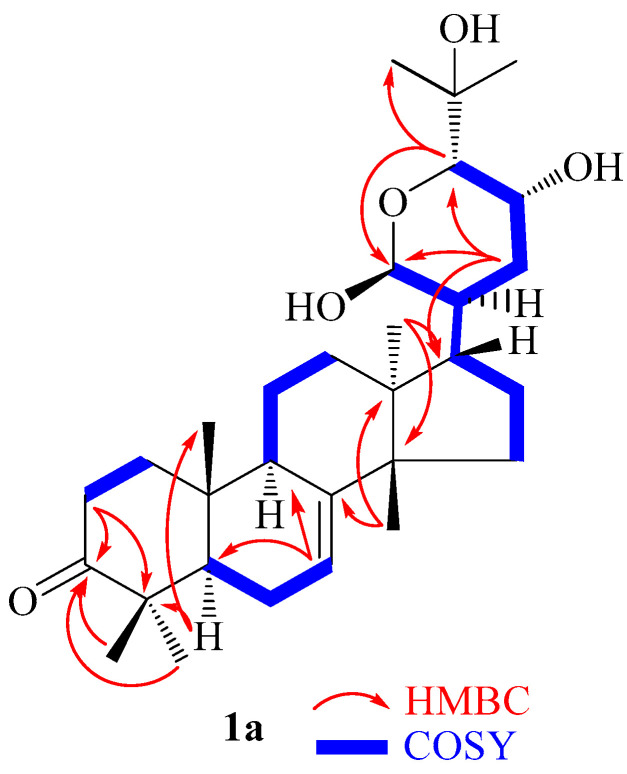
Important COSY and HMBC correlations of compound **1a**.

The HMBC (Table 1, Figure 2 and Figure S8a,b) spectrum revealed some important heteronuclear correlations that led to the construction of the skeleton of compound (**1abc**). Therefore, the correlation between the methyls Me-28/Me-29 (*δ*_C_/*δ*_H_ 25.1/1.03)/(*δ*_C_/*δ*_H_ 22.0/1.13) and the carbons at *δ*_C_ 219.2 (C-3), 53.9 (C-5) and 49.1 (C-4) led to the position of the ketone group and supported the triterpernoid skeleton [16,19,21]. However, the corrections between methyl Me-30 (*δ*_C_/*δ*_H_ 28.1/1.06) with carbons at *δ*_C_147.2 (C-8), 52.9 (C-14), and 35.9 (C-15) as well as methyl Me-18 (*δ*_C_/*δ*_H_ 28.1/1.06) with carbons at *δ*_C_ 31.1 (C-13), 44.9 (C-13), 48.1 (C-17), and 52.5 (C-14) led to positioning of the double bond and supported the tirucallan moiety of the compound (**1abc**) epimeric mixture [16,18,19,21]. The oxymethine protons H-21*α*/H-21*β* (*δ*_C_/*δ*_H_ 103.2/5.21)/(*δ*_C_/*δ*_H_ 94.1/5.21) correlating with carbons at *δ*_C_ 52.0 (C-17), 50.2/47.9 (C-20), 32.9 (C-22), and 78.3/77.0 (C-23) supported the presence of the 21*α/β*-melianodiol (**1b**/**1c**) epimer in the mixture [16,17,18,22]. Finally, the free oxygen bearing methine H-21 (*δ*_C_/*δ*_H_ 94.1/5.26) correlating with carbons at *δ*_C_ 73.6 (C-24) and 32.3 (C-22) agrees with the existence of free hydroxylation of the pyran ring of bourjotinolone A, which has not been reported previously. This pyranic ring is also supported by the COSY cross peak correlations between H-21 (*δ*_H_ 5.26), H-20 (*δ*_H_ 2.12), H-17 (*δ*_H_ 1.85), H-22 (*δ*_H_ 1.70/1.65), H-23 (*δ*_H_ 4.10), and H-24 (*δ*_H_ 3.60) [16]. The relative configurations of the chiral centers were deduced from the NOESY spectrum (Figure 2 and Figure S9) through homonuclear correlations between H-21 (*δ*_H_ 5.26) and H-20 (*δ*_H_ 2.12) and H-18 (*δ*_H_ 0.91), showing their *alpha*-orientation, while the correlation between H-22 (*δ*_H_ 4.11) and H-24 (*δ*_H_ 3.60) was *beta*-oriented. These special orientations were in agreement with the reported data of Xu et al. [16]. Therefore, compound **1** was established as an isomeric mixture including a stable new 21*β*-hydroxybourjotinolone A (**1a**), together with a mutarotant of 21*α*-melianodiol or 21,23-epoxy-21*α*,24,25-trihydroxytirucall-7-en-3-one (**1b**) [16,17,22] and 21*β*-melianodiol or 21,23-epoxy-21*β*,24,25-trihydroxytirucall-7-en-3- one (**1c**) [16,17,18] in the ratio of 3:1:1. Compounds 21*α*-melianodiol (**1b**) and 21*β*-melianodiol (**1c**) usually occur as an epimeric mixture due to the mutarotation formation at C-21, while the mixture of 21*α/β*-melianone and bourjotinolone A also occurs as a mixture of position isomers when they are not abundant in the plant species [17,19,23].

The remaining 13 known compounds were identified as follows: bellericagenin B (**2**) [24,25], alphitolic acid (**3**) [26,27], methyl angolensate (**4**) [28], rohituka-3 (**5**) [11,29], khayanolide E (**6**) [30,31], oleanolic acid (**7**) [32], Belamcanidin (**8**) [25,33], catechin (**9**) [34], gynuramide IV (**10**) [35], a mixture of *β*-sitosterol (**11**) and stigmasterol (**13**) [36], and a mixture of *β*-sitosterol glycoside (**12**) and stigmasterol glycoside (**14**) [37,38].

### 2.2. Antileishmanial, Antiplasmodial, and Cytotoxic Activities

**Figure 3:**
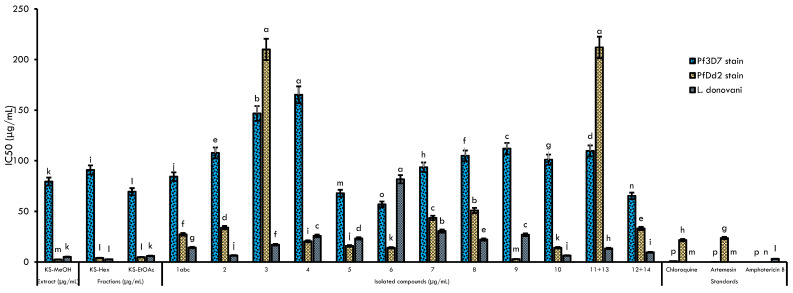
Column diagram summarizing the biological activities of the compounds, fractions, and extract from *Khaya senegalensis*. The results with the same letters are not significantly different (*p* < 0.05). The error bars represent the standard deviation of measurement of samples from duplicate runs.

The MeOH crude extract from *Khaya senegalensis* stem bark, the fractions, and the isolated compounds were evaluated for their antiplasmodial activity against chloroquine-resistant Dd2 and chloroquine-sensitive 3D7 strains of *Plasmodium falciparum* as well as antileishmanial and cytotoxicity activities against the *Leishmania donovani* strain and RAW 264.7 cancer cell lines, respectively (Figure 3, Table 2). According to a previously published report, the antiplasmodial activity of these samples was categorized as follows: high (IC_50_ < 5 μg/mL), promising (5 ≤ IC_50_ < 15 μg/mL), moderate (15 ≤ IC_50_ < 50 μg/mL), and weak or inactive (IC_50_ ≥ 50 μg/mL) [39]. The crude extract of *K. senegalensis* (**KS**-**MeOH**) exhibited high antiplasmodial activity against *Pf*Dd2 coupled with a good selectivity index (SI) toward RAW cells (IC_50_ = 2.5 ± 0.12 μg/mL, SI 19) and was inactive against *Pf*3D7 (IC_50_ = 79.4 ± 0.33 μg/mL, SI 0.59). Consequently, the crude extract was found to be highly active against *Leishmania donovani* promastigotes (SI value of 9.28) with a moderate SI in RAW cells (IC_50_ = 5.12 ± 0.7 μg/mL, SI 0.59). Liquid-liquid partitioning of **KS**-**MeOH** afforded two fractions (**KS**-**Hex** and **KS**-**EtOAc**) that displayed equipotent antiplasmodial (*Pf*3D7: IC_50_ 69.7–90.87 μg/mL^−^; *Pf*Dd2: IC_50_ 4.05–4.78 μg/mL), antileishmanial (IC_50_ 2.68–5.99 μg/mL^−^) and cytotoxic activities (SI_*Pf*3D7 0.72–0.88, SI_*Pf*3D7 10.59–10.93, SI Leish 8.45–30.12).

The purification of **KS**-**Hex** and **KS**-**EtOAc** led to the isolation of diverse classes of secondary metabolites. Table 2 also reports the biological potencies of the isolated compounds. The criteria for antiplasmodial activity were focused on the established limits [40], where IC_50_ < 1 μg/mL indicates that the compound has excellent activity; IC_50_ of 1–20 μg/mL indicates good activity; IC_50_ of 20–100 μg/mL indicates moderate activity; IC_50_ of 100–200 μg/mL indicates low activity; and IC_50_ > 200 μg/mL indicates inactivity. Overall, compounds **1abc**–**14** showed antiplasmodial activity with IC_50_ values ranging from 56.98 μg/mL (khayanolide E) (**6**) to 165.19 μg/mL (methylangolensate) (**4**) on *Pf*3D7 and 2.95 μg/mL (catechin) (**9**) to 50.97 μg/mL (belamcanidin) (**8**) on *Pf*Dd2 with catechin (**9**), khayanolide E (**6**), gyrunamide IV (**10**), rhohituka-3 (**5**) and methylangolensate (**4**) displaying the highest activity against *Pf*Dd2 (IC_50_ ˂ 20 μg/mL) with IC_50_ values of 2.95, 14.03, 14.13, 15.92, and 20.51 μg/mL, respectively. Alphitolic acid (**3**) and the mixture of *β*-sitosterol (**11**) and stigmasterol (**13**) were inactive against the multidrug-resistant *Pf*Dd2 strain (IC_50_ ˃ 200 μg/mL). The active compounds (**4**–**10**) showed resistance indices of less than 1, indicating a more pronounced potency against the resistant strain *Pf*Dd2 than against the sensitive strain. Concurrently, they were also found to be more selective toward *Pf*Dd2 (13.73 to 239.05) than the chloroquine-sensitive *Pf*3D7 strain (SI 0.24 to 10.71). These compounds (**1abc**–**14**) were also tested against the promastigote form of *L. donovani* (Table 2). Globally, the isolated compounds revealed moderate to high inhibition of *L. donovani* promastigotes (IC_50_: 6.5–81.73 μg/mL), and except for khayanolide E (**6**) (SI 0.99), all of the samples were selective toward RAW 264.7 cells.

## 3. Discussion

Parasitic diseases, including malaria and leishmaniasis, represent a public health challenge, particularly for poor populations living in developing countries. Although significant progress has been made through the development of control methods such as chemotherapy, the impact of these diseases remains in the countries concerned, causing enormous socioeconomic losses [41,42]. This is due in particular to the drawbacks associated with approved drugs, including toxicity, duration of treatment regimens, and the spreading resistance of parasites to first-line drugs [42,43]. For several decades, medicinal plants and natural products have played a key role in the discovery of lead compounds, which have been used to advance the discovery and development of drugs against infectious diseases. In fact, 20 antiparasitic agents were approved by the Food and Drug Administration between 1981 and 2019, among which nine (45%) were derived from natural products [44]. Moreover, in the case of malaria, the two famous compounds that have positively revolutionized the management of this disease are quinine and artemisinin isolated from *Cinchona sp*. stem bark and *Artemisia annua,* respectively [45,46]. This supports the hypothesis that remarkable discoveries can be made from medicinal plants for the identification of new scaffolds against infectious diseases, including malaria and leishmaniasis.

The goal of this study was to search for bioactive secondary metabolites from the stem bark of *K. senegalensis* (Meliaceae) that are endowed with antiplasmodial and antileishmanial activities. The methanol extract (**KS**-**MeOH**) of *K. senegalensis* showed moderate antiplasmodial activity against *Pf*3D7, a chloroquine-sensitive strain, but exhibited a more promising effect against multidrug-resistant *Pf*Dd2 and *Leishmania donovani* promastigotes (Table 2, Figure 3). A similar trend was observed with the Sudanese methanolic extract from *K. senegalensis* stem bark against *Pf*3D7 and *Pf*Dd2, with IC_50_ values of 25 μg/mL and 150 μg/mL, respectively [47]. Moreover, two other distinct studies have previously demonstrated the antiplasmodial activity of extracts from this plant species against *Pf*3D7 and *Pf*W2; however, the bioactive compounds were not reported [48,49]. With regard to its antileishmanial properties, very limited studies have reported the activity of *K. senegalensis* against *Leishmania* sp. parasites [50,51]. In addition, the extract **KS**-**MeOH** was shown to be selective against *Pf*Dd2 (SI > 19) and *L. donovani* (SI > 9.28) but not against *Pf*3D7 compared to the cytotoxic effect on RAW 264.7 cell lines. Climate change, geographical location, and harvesting period can modify the qualitative and quantitative content of secondary metabolites in medicinal plants and consequently their biological activities [52]. This further suggests that **KS**-**MeOH** preferentially inhibits *Pf*Dd2 and *Leishmania* parasites rather than mammalian cells. Recently, plants from the Meliaceae family have been recognized as a vast source of lead candidates for the development of antiplasmodial and insecticidal drugs [53]. Therefore, our phytochemical investigation of the antiprotozoal activity of *Khaya senegalensis* confirmed the activities previously reported [52]. The subsequently afforded hexane (**KS**-**Hex**) and ethyl acetate (**KS**-**EtOAc**) fractions from the **KS**-**MeOH** extract displayed the same antileishmanial, antiplasmodial, and selectivity profiles as the crude **KS**-**MeOH** extract. It can be alluded that the potent activity of **KS**-**MeOH** crude extract and fractions **KS**-**Hex** and **KS**-**EtOAc** (*Pf*Dd2 IC_50_ 2.5–4.78 μg/mL, SI 10.59–19.93; *L. donovani* IC_50_ 2.68–5.99 μg/mL, SI 8.45–30.12) is consequential of synergistic interactions between limonoids and eventually other secondary metabolites that are present in the extracts.

In further detail, a total of 16 compounds (**1abc**–**14**) were isolated from both **KS**-**Hex** and **KS**-**EtOAc** using several column chromatographic techniques, including a new ordered limonoid-type bourjotinolone (**1a**) along with triterpenoid-type limonoids (**1bc**, **4**–**6**). The protolimonoid compound (**1abc**) was found to be a mixture of a new limonoid 21*β*-hydroxybourjotinolone A (**1a**) together with an epimeric mixture of 21*α*-melianodiol (**1b**) and 21*β*-melianodiol (**1c**). However, degraded triterpenoid-type limonoids (compounds **4**–**6**), lupanes (**3**), oleanane (**7**) and steroids (**11**–**14**), two flavonoids (**8**–**9**) and one ceramide (**10**) were also identified here. Most of these compounds (**2**, **3**–**9** and **11**–**14**) have been previously reported from *K. senegalensis* [8,11]. Regarding their antiparasitic activities, as far as we know, the current study reveals for the first time the antiplasmodial and antileishmanial activities of alphitolic acid (**3**), rohituka-3 (**5**), belamcanidin (**8**), bellericagenin B (**2**), and gynuramide IV (**10**). Alphitolic acid (**3**) and Rohituka-3 (**5**) isolated from the same plant have been reported as antibacterial agents with cidal effects against *Escherichia coli* and *Pseudomonas aeruginosa* [11]. We have shown that, in addition to antibacterial activity, Rohituka-3 (**5**) has broad antiparasitic activity, while alphitolic acid (3) is mainly active against *Leishmania donovani*. For the other compounds, belamcanidin (**8**), bellericagenin B (**2**), and gynuramide IV (**10**), very little biological data are available in the literature. Our findings on the antiplasmodial activity of khayanolide (**6**) corroborate previous studies on this compound. Indeed, while investigating the antibacterial and antiplasmodial activity of constituents from the stem bark of *Trichilia monadelpha* (Thonn.), a plant from the Meliaceae family, Djoumessi and collaborators found that khayanolide (**6**) has a potent effect in vitro with an IC_50_ value of 1.68 μg/mL against the chloroquine-sensitive/sulfadoxine-resistant *Pf*3D7 strain [54].

Previously identified as an antibacterial agent [11], methylangolensate (**4**) was also identified here as an antileishmanial and antiplasmodial natural product. Oleanolic acid is well reputed for its antiplasmodial and antileishmanial activities. In fact, Cimanga et al. [55] and Peixoto et al. [56] have shown that it inhibits the growth of a clinical isolate of *P. falciparum* and *Leishmania amazonensis* with IC_50_ values of 15.2 μg/mL and 439.5 μg/mL, respectively [55,56]. Most importantly, Torres-Santos et al. [57] demonstrated that in addition to its antipromastigote effect (IC_50_ of 10 μg/mL), oleanolic acid from *Pourouma guianensis* also possesses antiamastigote inhibitory activity (IC_50_ of 27 μg/mL) [57]. Catechin (**9**) is a phenolic compound also known for its antiparasitic properties. Mogana et al. [58] investigated the antipromastigote activity of isolates from *Canarium patentinervium* and found an IC_50_ value of 478.93 ± 0.28 μg/mL for catechin [58]. Interestingly, two dimeric proanthocyanidins, namely, catechin-(4a,6)-catechin and catechin-(4a,8)-catechin), isolated from *Khaya senegalensis*, exhibited significant effects when tested against the amastigote forms of *Leishmania donovani,* with IC_50_ values of 3.85 and 3.98 μg/mL, respectively [51]. Moreover, catechin isolated from *Garcinia celebica* leaves was found to inhibit *Plasmodium falciparum* growth through the induction of oxidative stress [59]. Another study [60] demonstrated that at a concentration of 400 mg/kg, catechin from the leaf extract of *Osyris quadripartita* displayed an antimalarial effect with a suppression value of 64.26% [61].

Compounds **1b**, **1c**, and **10** are reported for the first time from the *Khaya* genus, while compound **1a** is an unprecedented compound and reported for the first time from the Meliaceae family. Compound **1** (**1abc**), a mixture of three limonoids (hydroxybourjotinolone A, 21*α*-melianodiol, and 21*β*-melianodiol), displayed moderate activity against *Pf*3D7 (IC_50_ 84.3 μg/mL), promising activity against both *Pf*Dd2 (IC_50_ 27.38 μg/mL), high activity against *Leishmania donovani* (IC_50_ 14.31 μg/mL), and high selectivity (SI > 10) for all strains. This is particularly relevant, as previous research has revealed antiplasmodial and antileishmanial activities for compound 7α-obacunyl acetate (IC_50_ 5.14 μg/mL) and trigilgianin (IC_50_ 6.044 μg/mL), which are two limonoids isolated from *Trichilia gilgiana* (Meliaceae) [62] and *Entandrophragma angolense* (Meliaceae), respectively [62]. Previous studies have shown that melanodiol is an anticancer [62], antioxidant, anti-inflammatory [63], and antibacterial agent [64]. The presence of such compounds with different properties could therefore explain the good activity profile obtained with this mixture. The results once again highlight the extensive antiparasitic properties of secondary metabolites belonging to the limonoid class. It is also important to point out that limonoids are reported to be the most abundant and best chemophenetic markers of the Meliaceae family as well as the *K. senegalensis* species [1,3,8,11,65]. This study therefore agrees with the classification of this plant species and improves knowledge of the classes of secondary metabolites found in *K. senegalensis* as well as their biological properties.

## 4. Materials and Methods

### 4.1. General Experimental Procedures

An alpha spectrometer (Brüker) spectrophotometer was used to record the IR spectrum in KBr. For NMR analysis, samples were first dissolved (85 μL) in their dissolving solvent before introduction into Eurisotop tubes (ref. D048T) and then analysis with Brüker Avance II+ at 500 MHz and 600 MHz equipped with a TCI Cryoprobe working under TopSpin 3.2.5. The mass spectrometry (ESI-MS, positive mode) of the samples was performed on a pneumatically assisted API (Atmospheric Pressure Ionization) mass spectrometer (Waters) type SYNAPT G2 HDMS. HRESIMS were measured with a TOF (Time-of-flight) analyzer. Silica gel (0.063–0.200 mm) was used as the stationary phase for CC (chromatographic column) for the fractionation and purification of compounds. TLC and UV (CN-6 UV, 254 nm & 365 nm) were used to check the purity of the isolated compounds. Ceric sulfate and 20% sulfuric acid were used to visualize the spotted samples on TLC after heating at approximately 90 °C. Iodine vapor was also used for visualization.

### 4.2. Plant Material

The stem bark of *Khaya senegalensis* was harvested on 15 September 2019, at Tchatibali Village (14°5′0″ E) in the Mayo-Danay Division of the Far-North Region of Cameroon. The plant material was taken to the National Herbarium of Cameroon, where it was identified in comparison with an available voucher specimen deposited under registration number 56853/HNC.

### 4.3. Extraction and Isolation

The fresh stem bark of *K. senegalensis* was collected and allowed to dry at room temperature, then powdered (7.5 kg) and extracted for 3 days (72 h) with 22 L MeOH, leading to 500.7 g of MeOH crude extract. A total of 275.0 g of the MeOH crude extract was subjected to liquid-liquid extraction to afford 15.6 g of *n*-hexane (**KS**-**Hex**), 160.8 g of EtOAc (**KS**-**EtOAc**), and 10.1 g of MeOH residue.

The *n*-hexane fraction (**KS**-**Hex**) (15.0 g) was fractionated by open CC (silica gel, gradient system n-hexane/EtOAc, 150 mL), leading to 195 fractions that were grouped into 5 principal fractions (A, B, C, D, and E) based on their TLC profile. Fraction A (5.2 g) was purified using over CC (silica gel, *n*-hexane/EtOAc, isocratic, 50 mL) to afford a mixture of compounds 11 and 13 (1.3 g, 9:1, *v*:*v*). Fraction C (0.5 g) was first fractionated using open CC (silica gel, *n*-hexane/EtOAc 7:3, isocratic, *v*:*v*, 50 mL), which led to two subfractions indexed C1 and C2. Subfraction C2 (100.4 mg) was further purified over Sephadex LH-20 (CH_2_Cl_2_/MeOH 1:1, isocratic, *v*:*v*, 5 mL), leading to a mixture of **1abc** (5.1 mg, 7:3, *v*:*v*). The main fractions D (3.0 g) and E (0.9 g) were also purified using open CC (silica gel, gradient system n-hexane/EtOAc, 50 mL) to afford compounds **5** (7 mg, 3:7, *v*:*v*) and **10** (2 mg, 0:10, *v*:*v*), respectively (Figure 1).

The EtOAc fraction (**KS**-**EtOAc**) (150.0 g) was fractionated by open CC (silica gel, *n*-hexane/EtOAc and EtOAc/MeOH, gradient, 150 mL), leading to 219 fractions that were grouped into 6 major fractions (F, G, H, I, J and K) based on their TLC profile. Fraction F (20.1 g) was further purified using open CC (silica gel, *n*-hexane/EtOAc, gradient, 50 mL) to afford compounds **8** (109.8 mg, 8:2, *v*:*v*) and **7** (2.1 g, 8.5:2.5, *v*:*v*). Fraction G (10.3 g) was also purified by open CC (silica gel, *n*-hexane/EtOAc, gradient, 50 mL), leading to **3** (72.1 mg, 6:4, *v*:*v*). Fraction H (40.5 g) was purified using open CC (silica gel, *n*-hexane/EtOAc, gradient, 50 mL) to afford compounds 4 (41.1 mg, 1:1, *v*:*v*) and **6** (82.3 mg, 1:1, *v*:*v*). Fraction I (30.7 g) was purified using open CC (silica gel, *n*-hexane/EtOAc, gradient, 50 mL) to afford compounds **9** (4.1 mg, 3:7, *v*:*v*) and **2** (6.1 mg, 3:7, *v*:*v*). Fraction J (10.3 g) was finally purified by open CC (silica gel, EtOAc/MeOH, isocratic, 50 mL) to afford a mixture of compounds **12** and **14** (1.3 g, 9:1, *v*:*v*).

### 4.4. Physicochemical Characteristics of Compounds **1abc**

*21β-hydroxybourjotinolone A* (**1a**), white powder (MeOH); UV: 268, 365, 405, 666 nm; IR: 3 669–3 200, 2 964–2 869, 1 697, 1 488, 1 468, 1 444, 1 270, 1 070, 970 cm^−1^; HR-ESI-MS [M + Na]^+^ *m/z* 511.3394 (calculated for C_30_H_48_NaO_5_^+^, 511.3399); ^1^H NMR (CD_3_OD, 600 MHz) *δ* 2.04 (1H, m, H1)/1.47 (1H, m, H1), 2.84 (1H, brtd, *J* = 14.6, 5.6 Hz, H2)/2.19 (1H, brd, *J* = 14.6 Hz, H2), 1.75 (1H, brdd, *J* = 11.5, 6.0 Hz, H5), 2.13 (1H, m, H6)/1.28 (1H, m, H6), 5.35 (1H, brq, *J* = 3.1 Hz, H7), 2.38 (1H, m, H9), 1.62 (2H, m, H11), 1.99 (1H, m, H12)/1.81 (1H, m, H12), 2.20 (1H, m, H15)/2.17 (1H, m, H15), 1.87 (1H, m, H16)/1.31 (1H, m, H16), 1.85 (1H, m, H17), 0.91 (3H, s, H19), 1.05 (3H, s, H19), 2.12 (1H, m, H_2_O), 5.26 (1H, brd, *J* = 2.8 Hz, H21), 1.70 (1H, m, H22)/1.65 (1H, m, H22), 4.10 (1H, brt, *J* = 3.6 Hz, H23), 3.60 (1H, brd, *J* = 1.5 Hz, H24), 1.24 (3H, s, H26), 1.27 (3H, s, H27), 1.03 (3H, s, H28), 1.13 (3H, s, H29), 1.06 (3H, s, H30); ^13^C NMR (CD_3_OD, 150 MHz) *δ* 39.7 (C1), 35.9 (C2), 219.2 (C3), 49.1 (C4), 53.9 (C5), 25.1 (C6), 119.2 (C7), 147.2 (C8), 49.9 (C9), 36.2 (C10), 19.3 (C11), 31.1 (C12), 44.9 (C13), 52.4 (C14), 35.9 (C15), 27.7 (C16), 48.1 (C17), 23.2 (C18), 13.1 (C19), 40.0 (C20), 94.1 (C21), 32.3 (C22), 67.1 (C23), 73.6 (C24), 73.9 (C25), 26.8 (C26), 27.3 (C27), 25.1 (C28), 22.0 (C29), 28.1 (C30).
*21α-Melianodiol* (**1b**) *and 21β-melianodiol* (**1c**); UV: 268, 365, 405, 666 nm; IR: 3669–3200, 2964–2869, 1697, 1488, 1468, 1444, 1270, 1070, 970 cm^−1^; HR-ESI-MS [M + Na]^+^ *m/z* 511.3394 (calculated for C_30_H_48_NaO_5_^+^, 511.3399); ^1^H- and ^13^C-NMR: see Table 1.

### 4.5. Biological Activities

#### 4.5.1. Parasite and Cell Culture

The parasite strains used in this study are continuously cultured at the Antimicrobial and Biocontrol Agents Unit of the University of Yaoundé I, and were graciously provided by BEI Resources (https://www.beiresources.org (accessed on 16 February 2019)). They comprise the *Leishmania donovani* strain (1S(MHOM/SD/62/1S), the multiresistant (*Pf*Dd2), and the chloroquine-sensitive (*Pf3*D7-(MRA-102)) strains of *Plasmodium falciparum*. RPMI 1640 media supplemented with 0.2% sodium bicarbonate (Sigma Aldrich, Billings, MT, USA), 0.5% Albumax II (Gibco), 1% hypoxanthine 100X (Gibco, Billings, MT, USA), 25 mM HEPES, and 0.04% gentamicin (Sigma Aldrich) was used to sustain *P. falciparum* strains in sterile petri dishes. Fresh red blood cells from healthy O^+^ human volunteers were also added to the medium. These cells were suspended at 4% hematocrit (*v*/*v*) and incubated at 37 °C in a humidified environment with 5% CO_2_. To regularly maintain the culture, fresh medium was added to the culture every day. Thin blood smears stained with 10% Giemsa were used to microscopically monitor cell-cycle transition and parasitemia under immersion oil.

A T-75 cm^2^ cell culture flask was filled with complete Medium 199 [M199 supplemented with 10% heat-inactivated fetal bovine serum (HIFBS) and 100 IU/mL penicillin/streptomycin (Sigma, Darmstadt, Germany)] and used to grow the promastigote forms of *Leishmania donovani*. Every 72 h, flasks were subcultured and incubated at 28 °C.

The murine macrophage RAW 264.7 cell line was grown in DMEM (Dulbecco’s modified Eagle’s medium) containing 1% penicillin-streptomycin, 1% nonessential amino acids (NEA), and 10% HIFBS at 37 °C, and 5% CO_2_ in a humidified environment. A reverse Etaluma fluorescence microscope was used to view the cells every day, and the growth media was replaced every 72 h. At 80% confluence, cells were isolated using 0.25% Trypsine-EDTA (Gibco, MT, USA).

#### 4.5.2. Sample Preparation for Biological Assays

In 100% dimethyl sulfoxide (DMSO, Sigma Aldrich), stock solutions of crude extracts, fractions, and compounds were prepared for final concentrations of 100 mg/mL, 50 mg/mL, and 20 mg/mL, respectively. Chloroquine phosphate (CQ) (Sigma Aldrich) and artemisinin (Art) (Sigma Aldrich) were used as antiplasmodial reference medicines, and were prepared at 10 mM in sterile distilled water and 100% DMSO, respectively. For antileishmanial tests, amphotericin B (10 mM in 100% DMSO, Sigma Aldrich) was included in the experiment as a positive control.

#### 4.5.3. Antiplasmodial Activity of Compounds, Fractions and Extracts

The antiplasmodial efficacy of the samples was evaluated using the SYBR Green I-based fluorescence assay, as previously reported by Smilkstein et al. [66], on a sorbitol-synchronized ring stage culture of *Plasmodium falciparum* (*Pf*Dd2 and *Pf3*D7 strains). The test relies on the development of intense fluorescence following SYBR green binding to the exposed DNA found in wells containing healthy cells. Ninety microliters of parasites prepared with 1% hematocrit and 2% parasitemia and 10 μL of test sample extracts, fractions, isolated chemicals, chloroquine, and artemisinin were each added in triplicate into wells of a flat-bottomed microplate. The final test concentration values for the samples varied from 50 μg/mL-0.08 μg/mL and 100 μg/mL-0.16 μg/mL for extract/fractions. Following a 72 h incubation at 37 °C, 100 μL of SYBR Green I solution prepared with lysis buffer (0.2 μL of 10,000 SYBR Green I per mL of lysis buffer) made of EDTA (5 mM), Tris (20 mM; pH 7.5), Triton X-100 (0.08%; *v*/*v*), and saponin (0.008%; *m*/*v*) was added to each well. Fluorescence measurements were taken using an Infinite M200 microplate reader (Tecan, MT, USA) at emission and excitation wavelengths of 538 and 485 nm, respectively.

#### 4.5.4. Antileishmanial Potency of Compounds, Fractions, and Extracts

The resazurin-based assay was used to evaluate the antileishmanial activity of the extract (100 μg/mL–0.16 μg/mL), major fractions (100 μg/mL–0.16 μg/mL), and isolated compounds (50 μg/mL–0.08 μg/mL) against the promastigote form of *Leishmania donovani* [67]. Briefly, 10 μL of inhibitors at various triplicate doses (as prescribed for the antiplasmodial assay) were added to 90 μL of promastigotes from a logarithmic phase culture at a density of 4.10^5^ parasites/mL. The plates were incubated for 28 h at 28 °C, and 10 μL of resazurin solution (Sigma, Darmstadt, Germany) was added. Amphotericin B (Sigma, Darmstadt, Germany) (10 μg–0.016 μg/mL) and 0.1% DMSO-treated wells were used as the negative and positive controls, respectively. After a further 44 h of incubation, plates were read using a Magelan Infinite M200 fluorescence plate reader (Tecan, Männedorf, Switzerland) at excitation and emission wavelengths of 530 and 590 nm, respectively.

#### 4.5.5. Cytotoxicity Assay

The murine macrophages, RAW 264.7 cells, were plated into a 96-well cell culture treated flat-bottomed plate (SARSTEDT, Inc., Newton, NC 28658, USA) at a density of 10 × 10^4^ cells/100 μL/well and incubated overnight to enable cell adhesion. A fresh complete medium containing serially diluted concentrations of crude extract, fractions, and compounds was used to replace the previous medium. The final assay concentration values for extracts/fractions and compounds varied from 400 μg/mL to 0.8 μg/mL and 100–0.16 μg/mL, respectively. Podophyllotoxin (Sigma-Aldrich, Munich, Germany) was used as a positive control at a maximum dose of 10 μM. After 48 h of incubation, 10 μL of resazurin solution (0.15 mg/mL dissolved in PBS) was added to each well, and the plates were incubated for an additional 4 h [68]. Fluorescence data were finally recorded with a Magellan Infinite M200 plate reader (Tecan, Germany) at excitation and emission wavelengths of 530 nm and 590 nm, respectively.

#### 4.5.6. Data Analysis for the Performed Assays

The resulting fluorescence readings were used to calculate the growth inhibition percentages relative to the negative control for each sample and for each of the tested doses. GraphPad Prism 8.0 software was used to generate concentration-response curves from the nonlinear regression fit to determine the median inhibitory or cytotoxic concentration (IC_50_/CC_50_). The resistance index (RI) for antiplasmodial tests was calculated as the ratio between the samples’ IC_50_ values for the chloroquine-sensitive strain 3D7 and the multiresistant strain Dd2 [69]. Inhibitors with an RI value below 1 were considered to act preferentially on the resistant strain. Based on each inhibitor’s antiparasitic efficacy (IC_50_) and cell toxicity (CC_50_), selectivity indices (SI) were calculated [SI = CC_50_ (RAW)/IC_50_ (Parasites)]. Samples were classified as poorly toxic to RAW cells if their SI values were greater than 10 [70].

## 5. Conclusions

The chemical investigation undertaken on the stem bark of *K. senegalensis* led to the isolation of a new limonoid named 21*β*-hydroxybourjotinolone A (**1a**) in a C-21 epimeric mixture of 21*α*-melianodiol (1b) and 21*β*-melianodiol (1c), together with 13 known compounds. Compounds **2**, **3**–**9**, and **11**–**14** have previously been reported from *K. senegalensis;* while **1b**, **1c**, and **10** are reported for the first time from the *Khaya* genus, and **1a** is reported for the first time from the Meliaceae family. The mixture of bourjotinolone (1a) and 21*α/β*-melianodiol (**1b**/**1c**) was found to possess cross activity against *L. donovani* and *P. falciparum*, and a good selectivity profile toward the parasitic strains. Other compounds displayed moderate to high antiparasitic activity and selectivity, qualifying them as interesting starting points for novel antiprotozoal agent discovery. Concurrently, it would be useful to investigate the mechanisms through which these natural products exert their antiprotozoal effects as well as the correlations between structural activity and pharmacokinetic properties.

## Figures and Tables

**Table 1:** ^13^C, ^1^H and HMBC NMR data (^1^H 600 MHz, ^13^C 150 MHz, CD_3_OD) of compound **1abc**.

N°	21*β* Hydroxybourjotinolone A 1a			21*α*-Melianodiol 1b		21*β*-Melianodiol 1c	
	*δ*_C_	*δ*_H_	HMBC	*δ*_C_	*δ*_H_	*δ*_C_	*δ*_H_
1	39.7	2.04 (1H, m) 1.47 (1H, m)		39.7	2.04 (1H, m) 1.47 (1H, m)	39.7	2.04 (1H, m) 1.47 (1H, m)
2	35.9	2.84 (1H, brtd, 14.6, 5.6) 2.19 (1H, brd, 14.6)	1, 3, 4, 10	35.9	2.84 (1H, brtd, 14.6, 5.6) 2.19 (1H, brd, 14.6)	35.9	2.84 (1H, brtd, 14.6, 5.6) 2.19 (1H, brd, 14.6)
3	219.2	-		219.2	-	219.2	-
4	49.1	-		49.1	-	49.1	-
5	53.9	1.75 (1H, brdd, 11.5, 6.0)	4, 8, 19	53.9	1.75 (1H, brdd, 11.5, 6.0)	53.9	1.75 (1H, brdd, 11.5, 6.0)
6	25.1	2.13 (1H, m) 1.28 (1H, m)	4, 8, 10	25.1	2.13 (1H, m) 1.28 (1H, m)	25.1	2.13 (1H, m) 1.28 (1H, m)
7	119.2	5.35 (1H, brq, 3.1)	5, 6, 9	119.2	5.35 (1H, brq, 3.1)	119.2	5.35 (1H, brq, 3.1)
8	147.2	-		147.2	-	147.2	-
9	49.9	2.38 (1H, m)		49.9	2.38 (1H, m)	49.9	2.38 (1H, m)
10	36.2	-		36.2	-	36.2	-
11	19.3	1.62 (2H, m)		19.3	1.62 (2H, m)	19.3	1.62 (2H, m)
12	31.1	1.99 (1H, m) 1.81 (1H, m)		31.1	1.99 (1H, m) 1.81 (1H, m)	31.1	1.99 (1H, m) 1.81 (1H, m)
13	44.9	-		44.9	-	44.9	-
14	52.4	-		52.4	-	52.4	-
15	35.9	2.20 (1H, m) 2.17 (1H, m)	17	35.9	2.20 (1H, m) 2.17 (1H, m)	35.9	2.20 (1H, m) 2.17 (1H, m)
16	27.7	1.87 (1H, m) 1.31 (1H, m)		27.7	1.87 (1H, m) 1.31 (1H, m)	27.7	1.87 (1H, m) 1.31 (1H, m)
17	48.1	1.85 (1H, m)		52.0	1.85 (1H, m)	52.0	1.85 (1H, m)
18	23.2	0.91 (3H, s)	12, 13, 14, 17		0.92 (3H, s)		0.89 (3H, s)
19	13.1	1.05 (3H, s)	1, 5, 9, 10	13.1	1.05 (3H, s)	13.1	1.05 (3H, s)
20	40.0	2.12 (1H, m)		50.2	2.11 (1H, m)	47.9	2.00 (1H, m)
21	94.1	5.26 (1H, brd, 2.8)	22, 24	103.2	5.21 (1H, brdd, 4.0, 1.8)	94.1	5.21 (1H, brdd, 4.0, 1.8)
22	32.3	1.70 (1H, m) 1.65 (1H, m)	17, 21, 24	32.9	1.99 (1H, m) 1.82 (1H, m)	32.9	1.99 (1H, m) 1.82 (1H, m)
23	67.1	4.10 (1H, brt, 3.6)	20	77.0	4.34 (1H, brddd, 10.8, 5.0, 2.3)	78.3	4.46 (1H, brddd, 9.3, 6.7, 1.8)
24	73.6	3.60 (1H, brd, 1.5)	21, 23, 25, 27	78.5	3.20 (1H, brd, 2.3)	78.5	3.14 (1H, brd, 1.8)
25	73.9	-		74.5	-	73.6	-
26	26.8	1.24 (3H, s)	24, 25, 27	25.6	1.21 (3H, s)	25.6	1.19 (3H, s)
27	27.3	1.27 (3H, s)	24, 25, 26	27.5	1.26 (3H, s)	27.5	1.23 (3H, s)
28	25.1	1.03 (3H, s)	3, 4, 5, 29	25.1	1.03 (3H, s)	25.1	1.03 (3H, s)
29	22.0	1.13 (3H, s)	3, 4, 5, 28	22.0	1.13 (3H, s)	22.0	1.13 (3H, s)
30	28.1	1.06 (3H, s)	8, 13, 15	28.1	1.06 (3H, s)	28.1	1.06 (3H, s)

*δ* (ppm), (br: broad, s: singlet, d: doublet, t: triplet, q: quadruplet, m: multiplet, *J* (Hz)), blue: for differences.

**Table 2:** Antileishmanial and antiplasmodial activities and selectivity of compounds, fractions, and crude extract from *K. senegalensis*.

Samples	Cytotoxicity on RAW 264.7 Cells	Antiplasmodial Activity (μg/mL), Selectivity (SI) and Resistance Index (RI)					Antileishmanial Activity (μg/mL) and Selectivity (SI)	
	CC_50_ ± SD	*Pf*3D7 IC_50_ ± SD	SI_*Pf*3D7	*Pf*Dd2 IC_50_ ± SD	SI_*Pf*Dd2	RI	IC_50_ ± SD	SI
**Extract (μg/mL)**								
**KS-MeOH**	47.52	79.40 ± 0.33	0.59	2.50 ± 0.12	19	0.03	5.12 ± 0.70	9.28
**Fractions (μg/mL)**								
**KS-Hex**	80.73	90.87 ± 0.08	0.88	4.05 ± 0.00	19.93	0.04	2.68 ± 0.42	30.12
**KS-EtOAc**	50.64	69.52 ± 0.33	0.72	4.78 ± 0.36	10.59	0.06	5.99 ± 0.77	8.45
**Isolated compounds (μg/mL)**								
**21*β*-hydroxylbourjotinolone A (1a) + 21*α*-melianodiol (1b) + 21*β*-melianodiol (1c)**	>400	84.30 ± 0.11	>4.74	27.38 ± 0.18	>14.6	0.32	14.31 ± 0.87	>27.94
**Bellericagenin B (2)**	>400	107.72 ± 0.37	>3.81	34.02 ± 0.09	>11.75	0.31	6.5 ± 0.01	>61.54
**Alphitolic acid (3)**	35.76	146.77 ± 0.13	0.24	>200	ND	ND	17.09 ± 0.90	2.09
**Methylangolensate (4)**	72.74	165.19 ± 0.06	0.44	20.51 ± 0.45	34.13	0.12	25.83 ± 0.27	2.81
**Rohituka-3 (5)**	>400	68 ± 0.02	>5.88	15.92 ± 0.33	>25.13	0.23	23.35 ± 0.21	>17.13
**Khayanolide E (6)**	81.61	56.98 ± 0.08	1.43	14.03 ± 0.06	49.88	0.24	81.73 ± 0.12	0.99
**Oleanolic acid (7)**	>400	93.58 ± 0.92	˃4.27	43.58 ± 0.73	˃9.17	0.47	30.63 ± 0.67	˃13.06
**Belamcanidin (8)**	>400	104.92 ± 0.39	>3.82	50.97 ± 0.37	>7.84	0.48	22.21 ± 0.08	>18.013
**Catechin (9)**	>400	111.96 ± 0.04	>3.57	2.93 ± 0.02	>136.6	0.02	26.97 ± 0.70	>14.83
**Gynuramide IV (10)**	>400	101.07 ± 0.01	>3.96	14.13 ± 0.03	>28.3	0.14	6.43 ± 0.06	>62.22
***β*-sitosterol (11) + stigmasterol (13)**	>400	109.69± 1.66	>6.12	>200	ND	>1.1	13.47 ± 0.74	>29.69
***β*-sitosterol glycoside (12) + stigmasterol glycoside (14)**	>400	65.34 ± 0.89	˃3.65	33.16 ± 0.43	˃12	0.51	9.60 ± 0.64	˃41.65
**Reference drugs (μg/mL)**								
**Chloroquine**	-	0.022 ± 0.25	-	0.085 ± 0.01	-	-	-	ND
**Artemesin**	-	0.02 ± 0.00	-	0.024 ± 0.24	-	-	-	-
**Amphotericin B**	-	-	-	-	-	-	3.14 ± 0.46	-
**Podophyllotoxin**	0.71 ± 0.20	-	-	-	-	-	-	-

**KS**-**MeOH**: methanol extract of *K. senegalensis*, **KS**-**Hex**: *n*-hexane fraction of *Khaya senegalensis* extract, **KS**-**EtOAc**: ethyl acetate fraction of *K. senegalensis* extract, SI: selectivity index, RI: reactivity index, ND: not determined, NC: not calculated, *P. falciparum:* IC_50_: 50% inhibitory concentration in μg/mL; CC_50_: 50% cytotoxic concentration in μg/mL. The results are expressed as the mean ± standard deviation; the SI (selective index) of bioactive compounds was determined as a measure of their toxicity against RAW cell line macrophages. SI = CC_50_ against macrophages/IC_50_ against promastigotes.

## Data Availability

Data are contained within the article and Supplementary Materials.

## Supplementary Materials

The following supporting information can be downloaded at: https://www.mdpi.com/article/10.3390/molecules28207227/s1. Figure S1: HR-ESI-MS (+), [M + Na]^+^ of compound **1abc**; Figure S2: UV spectrum of compound **1abc**; Figure S3: IR spectrum (KBr) of compound **1abc**; Figure S4: ^1^H-NMR (CD_3_OD, 600 MHz) spectrum of compound **1abc**; Figure S5a: COSY (CD_3_OD) expanded spectrum of compound **1abc**; Figure S5b: COSY (CD_3_OD) full spectrum of compound **1abc**; Figure S6a: ^13^C-NMR (CD_3_OD, 150 MHz) expanded spectrum of compound **1abc**; Figure S6b: ^13^C-NMR (CD_3_OD, 150 MHz) full spectrum of compound **1abc**; Figure S7a: HSQC (CD_3_OD) expanded spectrum of compound **1abc**; Figure S7b: HSQC (CD_3_OD) full spectrum of compound **1abc**; Figure S8a: HMBC (CD_3_OD) expanded spectrum of compound **1abc**; Figure S8b: HMBC (CD_3_OD) expanded spectrum of compound **1abc**; Figure S9: NOESY (CD_3_OD) full spectrum of compound **1abc**; Figure S10: ^1^H-NMR (600 MHz, DMSO-d6) spectrum of compound **2**; Figure S11: ^13^C-NMR (150 MHz, DMSO-d6) spectrum of compound **2**; Figure S12: ^1^H-NMR (500 MHz, CD_3_OD) spectrum of compound **3**; Figure S13: ^13^C-NMR (125 MHz, CD_3_OD) spectrum of compound **3**; Figure S14: ^1^H-NMR (500 MHz, CDCl_3_) spectrum of compound **4**; Figure S15: ^13^C-NMR (125 MHz, CDCl_3_) spectrum of compound **4**; Figure S16: ^1^H-NMR (500 MHz, CDCl_3_) spectrum of compound **5**; Figure S17: ^13^C-NMR (125 MHz, CDCl_3_) spectrum of compound **5**; Figure S18: ^1^H-NMR (500 MHz, CDCl_3_) spectrum of compound **6**; Figure S19: ^13^C-NMR (125 MHz, CDCl_3_) spectrum of compound **6**; Figure S20: ^1^H-NMR (600 MHz, CD_3_OD) spectrum of compound **7**; Figure S21: ^13^C-NMR (150 MHz, CD_3_OD) spectrum of compound **7**; Figure S22: ^1^H-NMR (600 MHz, CDCl_3_) spectrum of compound **8**; Figure S23: ^13^C-NMR (150 MHz, CDCl_3_) spectrum of compound **8**; Figure S24: ^1^H-NMR (600 MHz, CDCl_3_) spectrum of compound **9**; Figure S25: ^13^C-NMR (150 MHz, CDCl_3_) spectrum of compound **9**; Figure S26: ^1^H-NMR (600 MHz, Pyridine-d_5_) spectrum of compound **10**; Figure S27: ^13^C-NMR (150 MHz, Pyridine-d_5_) spectrum of compound **10**; Figure S28: ^1^H-NMR (CDCl_3_, 500 MHz) spectrum of the mixture of compounds **11** and **13** and Figure S29: ^1^H-NMR (CDCl_3_, 500 MHz) spectrum of the mixture of compounds **12** and **14**.

## Sample Availability

Samples of the compounds are not available from the authors.
