# Supplementary material for: Antiplasmodial and Antileishmanial Activities of a New Limonoid and Other Constituents from the Stem Bark of Khaya senegalensis
Source: Molecules. 2023 Oct 23;28(20):7227. doi: 10.3390/molecules28207227 (PMC10609173; doi:10.3390/molecules28207227)
Supplement: Supplementary file 1 [file molecules-28-07227-s001.zip › molecules-2670929-supplementary.pdf]

molecules

Supplementary Information

Antiplasmodial and Antileishmanial Activities of a New Limonoid and Other Constituents from the Stem Bark of *Khaya senegalensis*

Gabrielle Ange Amang à Ngnoung [1], Yves Oscar Nganso Ditchou [1,*], Peron Bosco Leutcha [1,2], Darline Dize [3], Simplice Joël Ndendoung Tatsimo [2], Lauve Rachel Yamthe Tchokouaha [4], Theodora Kopa Kowa [5], Babalwa Tembeni [6], Hamadou Mamoudou [7], Madan Poka [6], Patrick Hulisani Demana [6], Xavier Siwe Noundou [6,*], Fabrice Fekam Boyom [3,4,8] and Alain Meli Lannang [2,9]

[1] Department of Chemistry, Faculty of Science, University of Maroua, Maroua P.O. Box 814, Cameroon; gabrielleangeamang@gmail.com (G.A.A.à.N.); peron.leutcha@gmail.com (P.B.L.)
[2] Natural Product and Environmental Chemistry Group (NAPEC), Department of Chemistry, Higher Teachers' Training College, University of Maroua, Maroua P.O. Box 55, Cameroon; jtatsimo@yahoo.com (S.J.N.T.); alainmeli@yahoo.com (A.M.L.)
[3] Antimicrobial and Biocontrol Agents Unit (AmBcAU), Laboratory for Phytobiochemistry and Medicinal Plants Studies, Department of Biochemistry, Faculty of Science, University of Yaoundé I, Yaoundé P.O. Box 812, Cameroon; darline.dize@yahoo.fr (D.D.); fabrice.boyom@fulbrightmail.org (F.F.B.)
[4] Laboratory of Pharmacology, Centre for Research on Medicinal Plants and Traditional Medicine, Institute of Medical Research and Medicinal Plants Studies, Yaoundé P.O. Box 13033, Cameroon; yamthe_lauve@yahoo.fr
[5] Laboratory of Phytochemistry, Centre for Research on Medicinal Plants and Traditional Medicine, Institute of Medical Research and Medicinal Plants Studies, Yaoundé P.O. Box 13033, Cameroon; kothera81@yahoo.fr
[6] Department of Pharmaceutical Sciences, School of Pharmacy, Sefako Makgatho Health Sciences University, Pretoria 0204, South Africa; btembeni2@gmail.com (B.T.); madan.poka@smu.ac.za (M.P.); patrick.demana@smu.ac.za (P.H.D.)
[7] Department of Biological Sciences, Faculty of Science, University of Maroua, Maroua P.O. Box 814, Cameroon; mamoudhmd@gmail.com
[8] Advanced Research and Health Innovation Hub (ARHIH), Yaoundé P.O. Box 20133, Cameroon
[9] Department of Chemical Engineering, School of Chemical Engineering and Mineral Industries, University of Ngaoundere, Ngaoundere P.O. Box 454, Cameroon
* Correspondence: nganso_yves@yahoo.fr (Y.O.N.D.); xavier.siwenoundou@smu.ac.za (X.S.N.); Tel.: +237-699-461-194 (Y.O.D.N.); +27-12-521-5647 (X.S.N.)

Citation: Ngnoung, G.A.A.À.; Ditchou, Y.O.N.; Leutcha, P.B.; Dize, D.; Tatsimo, S.J.N.; Tchokouaha, L.R.Y.; Kowa, T.K.; Tembeni, B.; Mamoudou, H.; Poka, M.; et al. Antiplasmodial and Antileishmanial Activities of a New Limonoid and Other Constituents from the Stem Bark of *Khaya senegalensis*. *Molecules* **2023**, *28*, x. https://doi.org/10.3390/xxxxx

Academic Editors: Francesca Mancianti and Valentina Virginia Ebani

Received: 3 October 2023
Revised: 19 October 2023
Accepted: 19 October 2023
Published: 23 October 2023

Copyright: © 2023 by the authors. Submitted for possible open access publication under the terms and conditions of the Creative Commons Attribution (CC BY) license (https://creativecommons.org/licenses/by/4.0/).

Abstract: *Plasmodium falciparum* and *Leishmania* sp. resistance to antiparasitic drugs has become a major concern in malaria and leishmaniasis control. These diseases are public health problems with significant socioeconomic impacts, and mostly affect disadvantaged populations living in remote tropical areas. This challenge emphasizes the need to search for new chemical scaffolds that preferably possess novel modes of action to contribute to antimalarial and antileishmanial research programs. This study aimed to investigate the antimalarial and antileishmanial properties of a methanol extract (**KS-MeOH**) of the stem bark of the Cameroonian medicinal plant *Khaya senegalensis* and its isolated compounds. The purification of **KS-MeOH** led to the isolation of a new ordered limonoid derivative, 21β-hydroxybourjotinolone A (**1a**), together with 15 known compounds (**1bc–14**) using a repeated column chromatography. Compound **1a** was obtained in an epimeric mixture of 21α-melianodiol (**1b**) and 21β-melianodiol (**1c**). Structural characterization of the isolated compounds was achieved with HRMS, and 1D- and 2D-NMR analyses. The extracts and compounds were screened using pre-established in vitro methods against synchronized ring stage cultures of the multidrug-resistant Dd2 and chloroquine-sensitive/sulfadoxine-resistant 3D7 strains of *Plasmodium falciparum* and the promastigote form of *Leishmania donovani* (1S(MHOM/SD/62/1S). In addition, the samples were tested for cytotoxicity against RAW 264.7 macrophages. Positive controls

consisted of artemisinin and chloroquine for *P. falciparum*, amphotericin B for *L. donovani*, and podophyllotoxin for cytotoxicity against RAW 264.7 cells. The extract and fractions exhibited moderate to potent antileishmanial activity with 50% inhibitory concentrations (IC_{50}) ranging from 5.99 ± 0.77 to 2.68 ± 0.42 μg/mL, while compounds displayed IC_{50} values ranging from 81.73 ± 0.12 to 6.43 ± 0.06 μg/mL. They were weakly active against the chloroquine-sensitive/sulfadoxine-resistant *Pf*3D7 strain but highly potent toward the multidrug-resistant *Pf*Dd2 (extracts, IC_{50} 2.50 ± 0.12 to 4.78 ± 0.36 μg/mL; compounds IC_{50} 2.93 ± 0.02 to 50.97 ± 0.37 μg/mL) with selectivity indices greater than 10 (SI_{Dd2} > 10) for the extract and fractions and most of the derived compounds. Of note, the limonoid mixture [21*β*-hydroxylbourjotinolone A (**1a**) + 21*α*-melianodiol (**1b**) + 21*β*-melianodiol (**1c**)] exhibited moderate activity against *P. falciparum* and *L. donovani.* This novel antiplasmodial and antileishmanial chemical scaffold qualifies as a promising starting point for further medicinal chemistry-driven development of a dually active agent against two major infectious diseases affecting humans in Africa.

Keywords: Meliaceae; *Khaya senegalensis*; limonoids; antileishmanial activity; antiplasmodial activity

Contents of Additional Information

Figure S1: HR-ESI-MS (+), $[M+Na]^+$ of compound **1abc**..... **Error! Bookmark not defined.**

Figure S2: UV spectrum of compound **1abc** .... **Error! Bookmark not defined.**

Figure S3: IR spectrum (KBr) of compound **1abc**................ **Error! Bookmark not defined.**

Figure S4: ^{1}H-NMR (CD_3OD, 600 MHz) spectrum of compound **1abcError! Bookmark not defined.**

Figure S5a: COSY (CD_3OD) expanded spectrum of compound **1abcError! Bookmark not defined.**

Figure S5b: COSY (CD_3OD) full spectrum of compound **1abcError! Bookmark not defined.**

Figure S6a: ^{13}C-NMR (CD_3OD, 150 MHz) expanded spectrum of compound **1abcError! Bookmark not defined.**

Figure S6b: ^{13}C-NMR (CD_3OD, 150 MHz) full spectrum of compound **1abcError! Bookmark not defined.**

Figure S7a: HSQC (CD_3OD) expanded spectrum of compound **1abcError! Bookmark not defined.**

Figure S7b: HSQC (CD_3OD) full spectrum of compound **1abcError! Bookmark not defined.**

Figure S8a: HMBC (CD_3OD) expanded spectrum of compound **1abcError! Bookmark not defined.**

Figure S8b: HMBC (CD_3OD) expanded spectrum of compound **1abcError! Bookmark not defined.**

Figure S9: NOESY (CD_3OD) full spectrum of compound **1abcError! Bookmark not defined.**

Figure S10: ^{1}H-NMR (600 MHz, DMSO-d_6) spectrum of compound **2Error! Bookmark not defined.**

Figure S11: ^{13}C-NMR (150 MHz, DMSO-d_6) spectrum of compound **2Error! Bookmark not defined.**

Figure S12: ^{1}H-NMR **(**500 MHz, CD_3OD**)** spectrum of compound **3Error! Bookmark not defined.**

Figure S13: ^{13}C-NMR (125 MHz, CD_3OD) spectrum of compound **3Error! Bookmark not defined.**

Figure S14: ^{1}H-NMR **(**500 MHz, $CDCl_3$**)** spectrum of compound **4Error! Bookmark not defined.**

Figure S15: ^{13}C-NMR (125 MHz, $CDCl_3$) spectrum of compound **4Error! Bookmark not defined.**

Figure S16: ^{1}H-NMR **(**500 MHz, $CDCl_3$**)** spectrum of compound **5Error! Bookmark not defined.**

Figure S17: ^{13}C-NMR (125 MHz, $CDCl_3$) spectrum of compound **5Error! Bookmark not defined.**

Figure S18: ^{1}H-NMR **(**500 MHz, $CDCl_3$**)** spectrum of compound **6Error! Bookmark not defined.**

Figure S19: ^{13}C-NMR (125 MHz, $CDCl_3$) spectrum of compound **6Error! Bookmark not defined.**

Figure S20: ^{1}H-NMR **(**600 MHz, CD_3OD**)** spectrum of compound **7Error! Bookmark not defined.**

Figure S21: ^{13}C-NMR (150 MHz, CD_3OD) spectrum of compound **7Error! Bookmark not defined.**

Figure S22: ^{1}H-NMR **(**600 MHz, $CDCl_3$**)** spectrum of compound **8Error! Bookmark not defined.**

Figure S23: ^{13}C-NMR (150 MHz, $CDCl_3$) spectrum of compound **8Error! Bookmark not defined.**

Figure S24: ^{1}H-NMR **(**600 MHz, $CDCl_3$**)** spectrum of compound **9Error! Bookmark not defined.**

Figure S25: ^{13}C-NMR (150 MHz, $CDCl_3$) spectrum of compound **9Error! Bookmark not defined.**

Figure S26: ^{1}H-NMR **(**600 MHz, Pyridine-d_5**)** spectrum of compound **10Error! Bookmark not defined.**

Figure S27: ^{13}C-NMR (150 MHz, Pyridine-d_5) spectrum of compound **10Error! Bookmark not defined.**

Figure S28: ^{1}H-NMR ($CDCl_3$, 500 MHz) spectrum of the mixture of compounds **11** and **13**.**Error! Bookmark not defined.**

Figure S29: ^{1}H-NMR ($CDCl_3$, 500 MHz) spectrum of the mixture of compounds **12** and **14**.**Error! Bookmark not defined.**

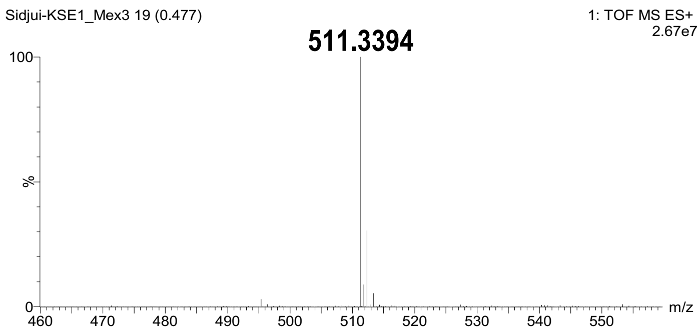

Figure S1. HR-ESI-MS (+), [M+Na]+ of compound **1abc**

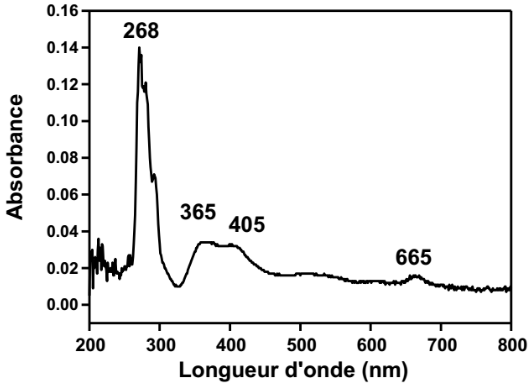

Figure S2. UV spectrum of compound **1abc**

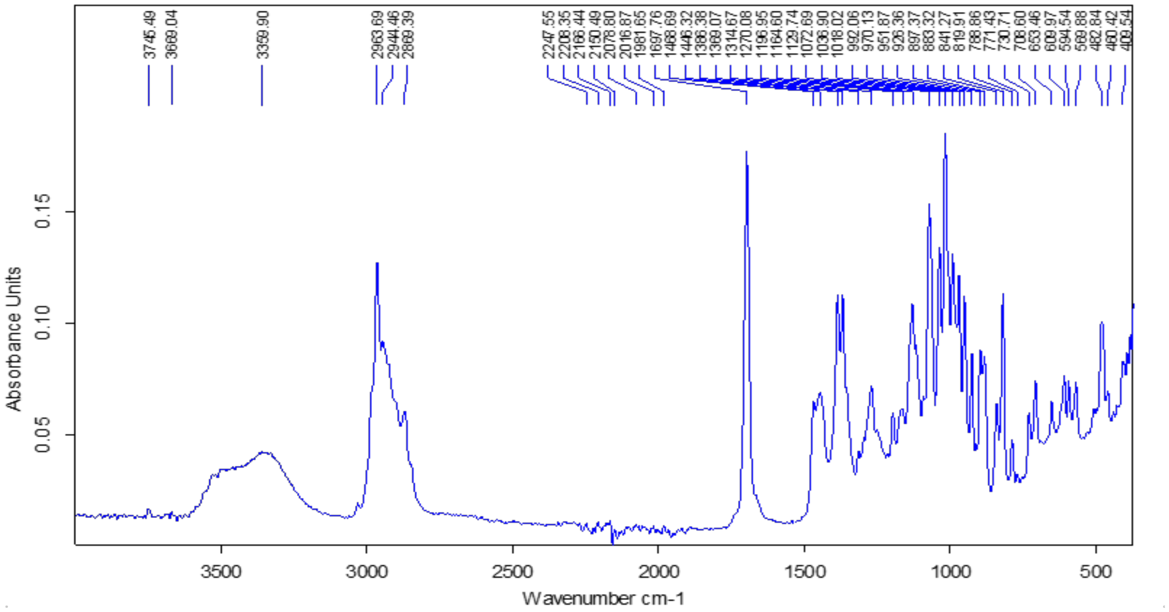

Figure S3. IR spectrum (KBr) of compound **1abc**

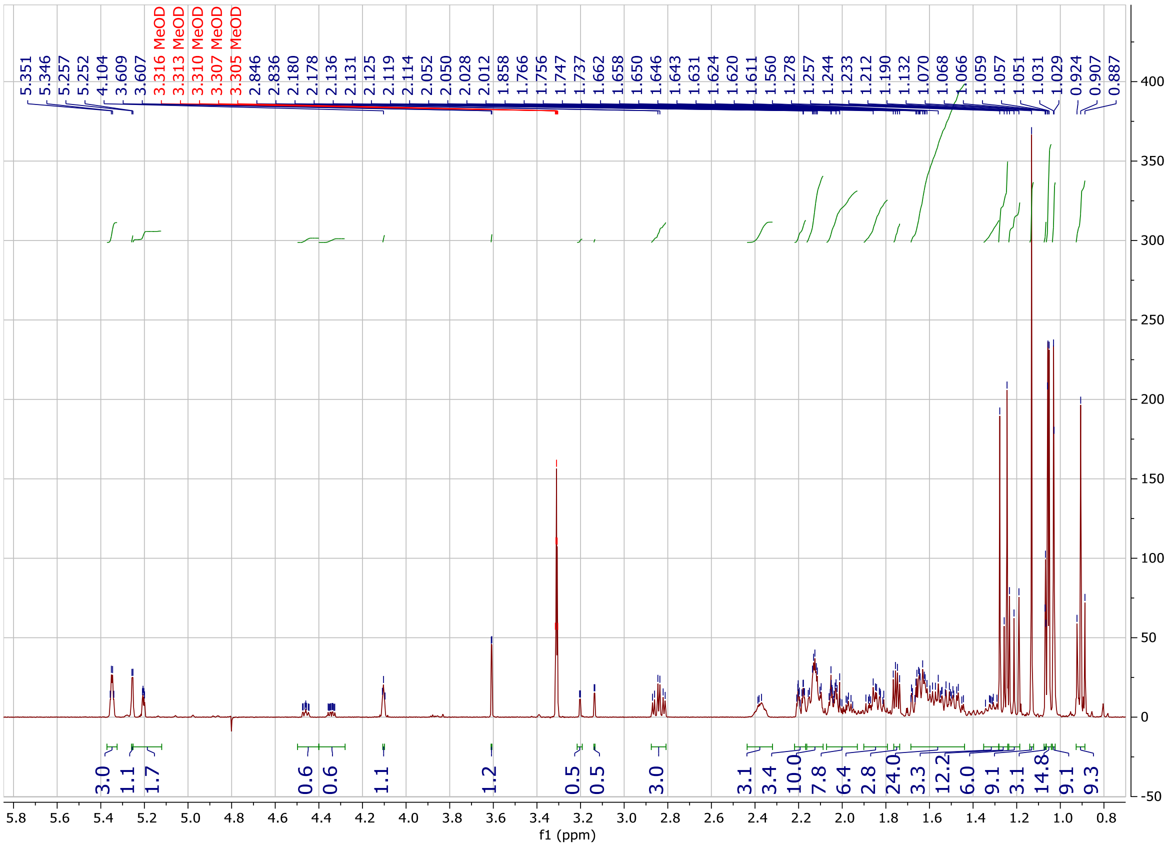

Figure S4. ^1H-NMR (CD_3OD, 600 MHz) spectrum of compound **1abc**

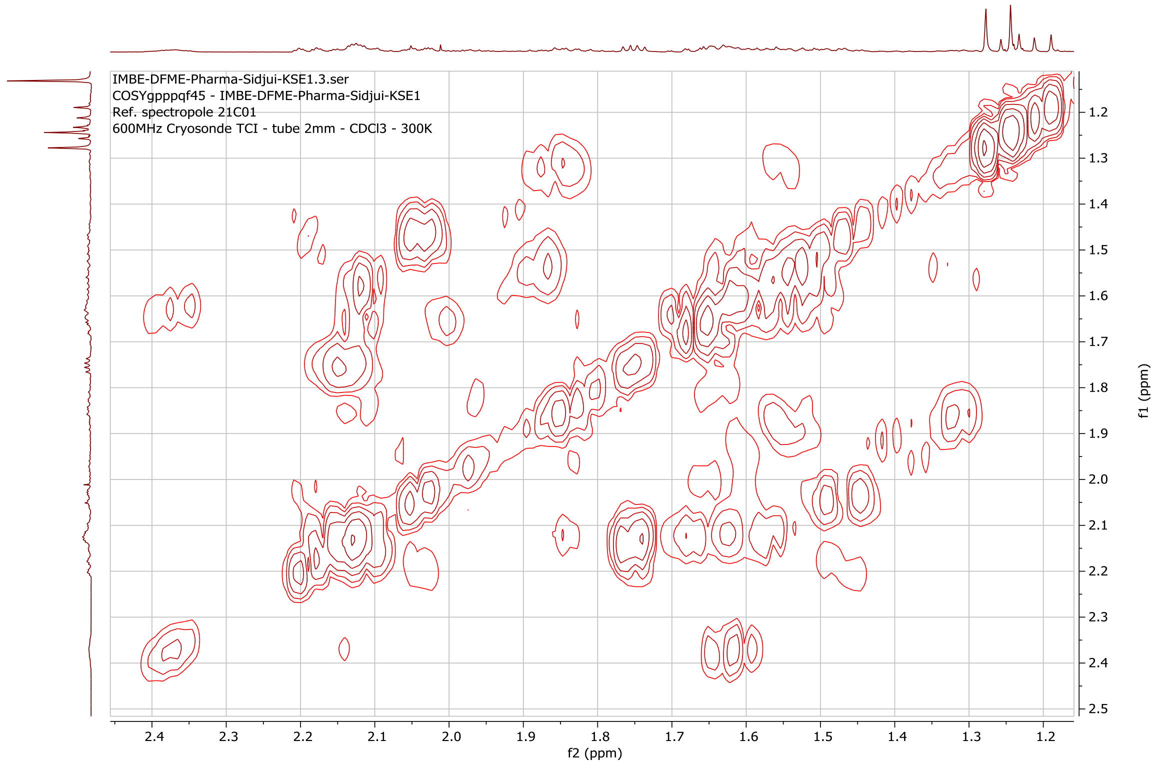

Figure S5a. COSY (CD_3OD) expanded spectrum of compound **1abc**

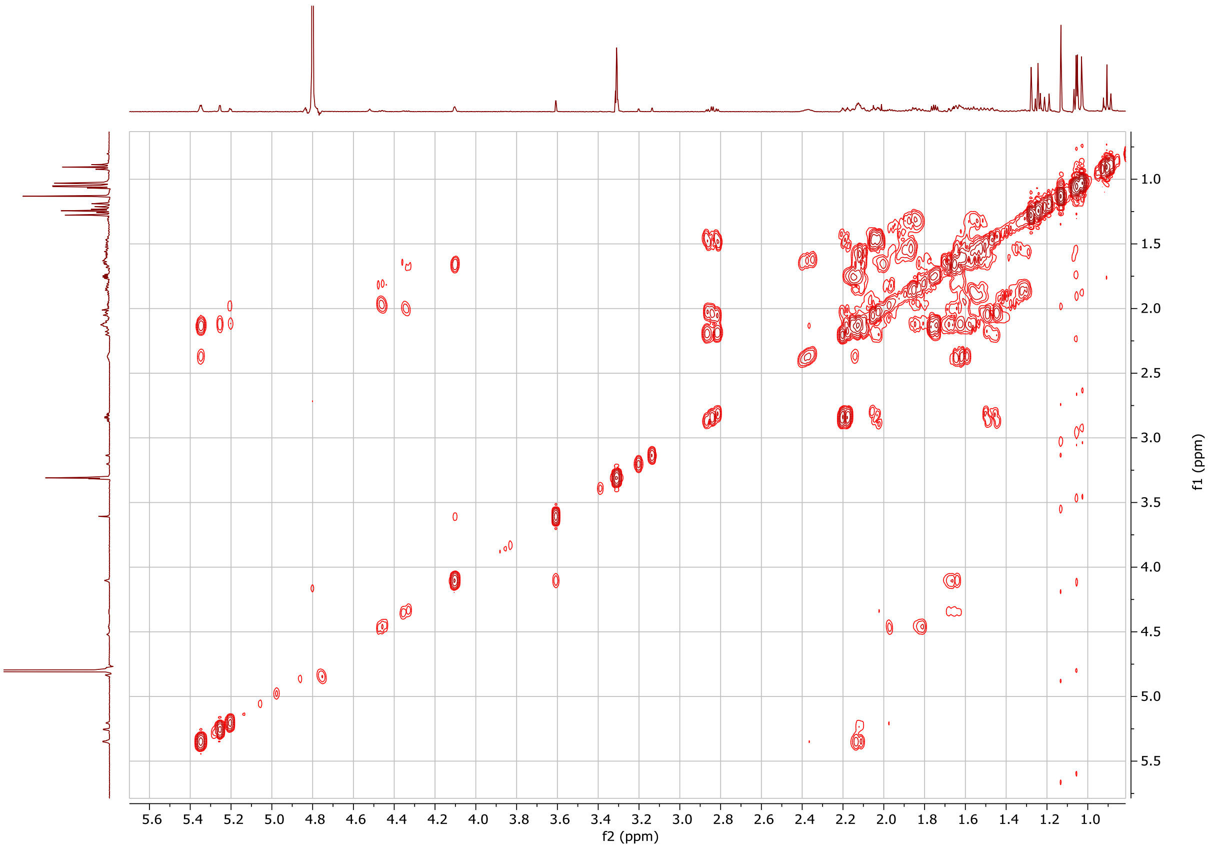

Figure S5b. COSY (CD_3OD) full spectrum of compound **1abc**

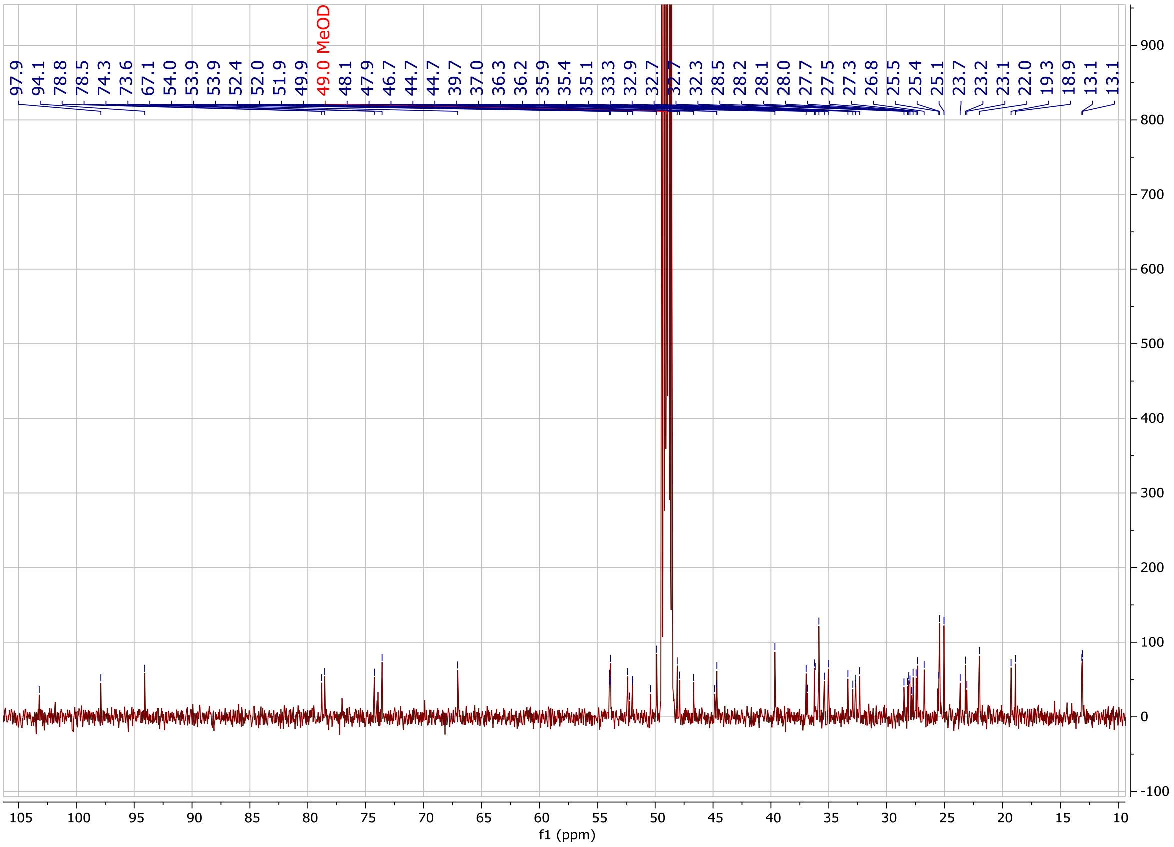

Figure S6a. ^{13}C-NMR (CD_3OD, 150 MHz) expanded spectrum of compound **1abc**

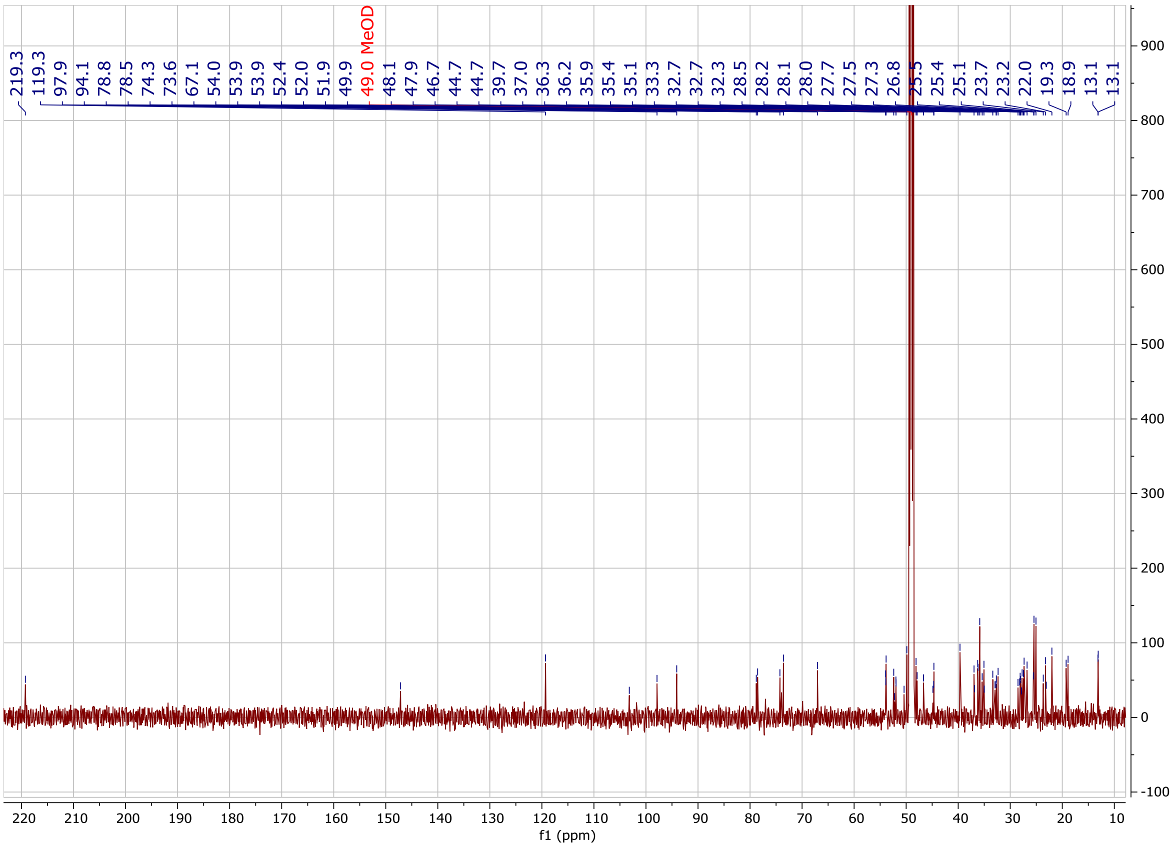

Figure S6b. ^{13}C-NMR (CD_3OD, 150 MHz) full spectrum of compound **1abc**

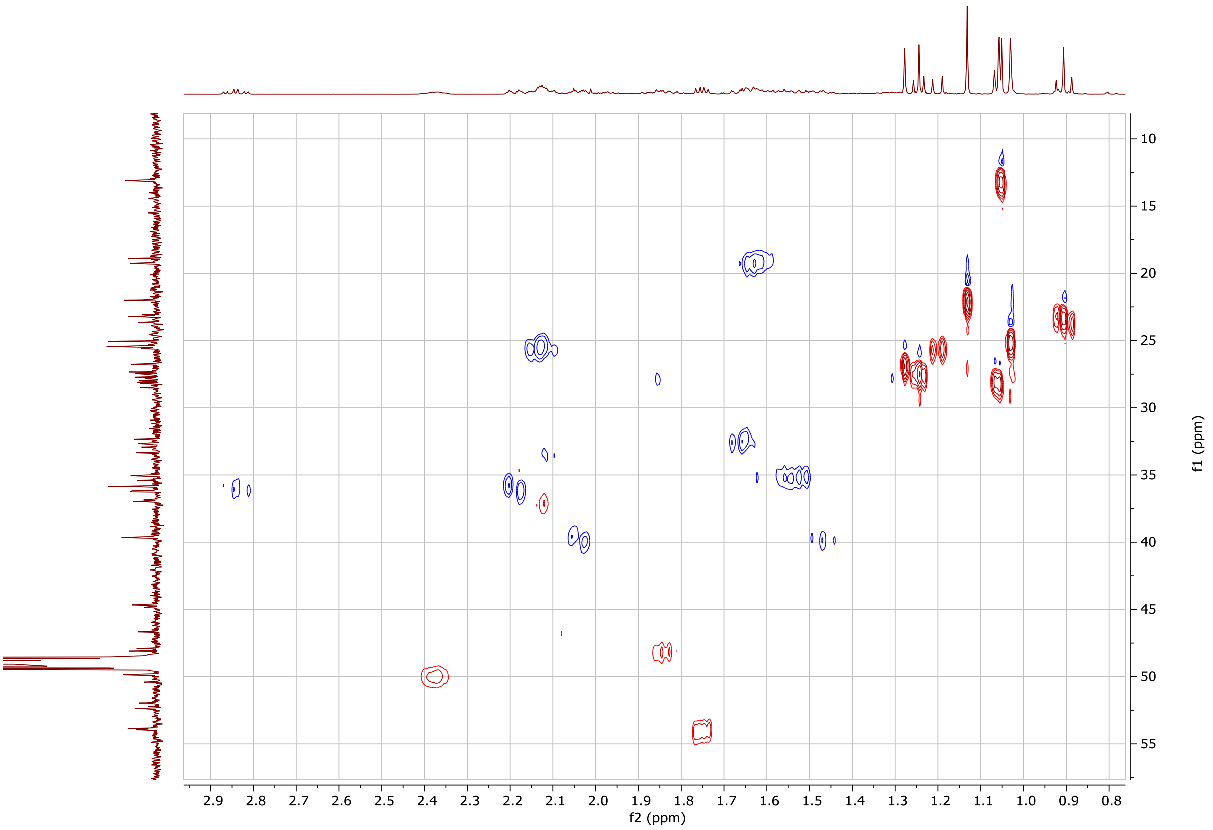

Figure S7a. HSQC (CD_3OD) expanded spectrum of compound **1abc**

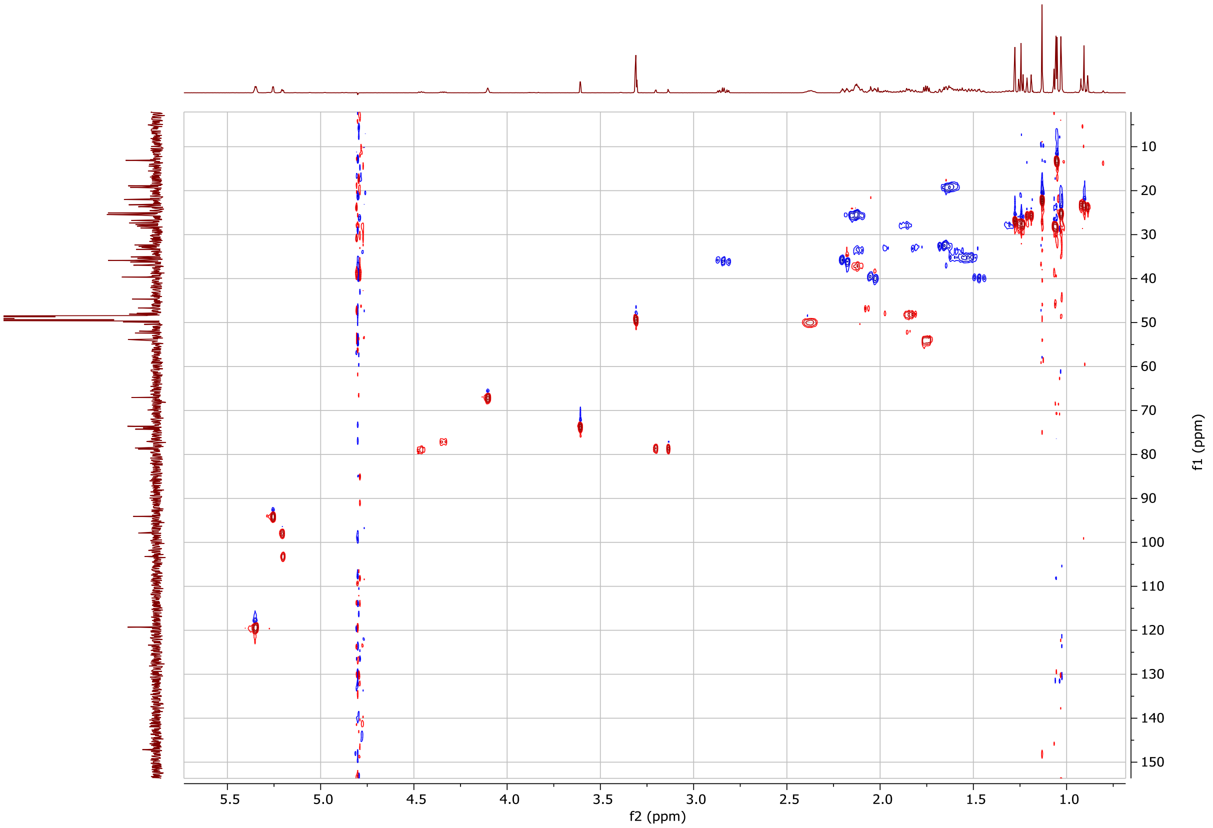

Figure S7b. HSQC (CD_3OD) full spectrum of compound **1abc**

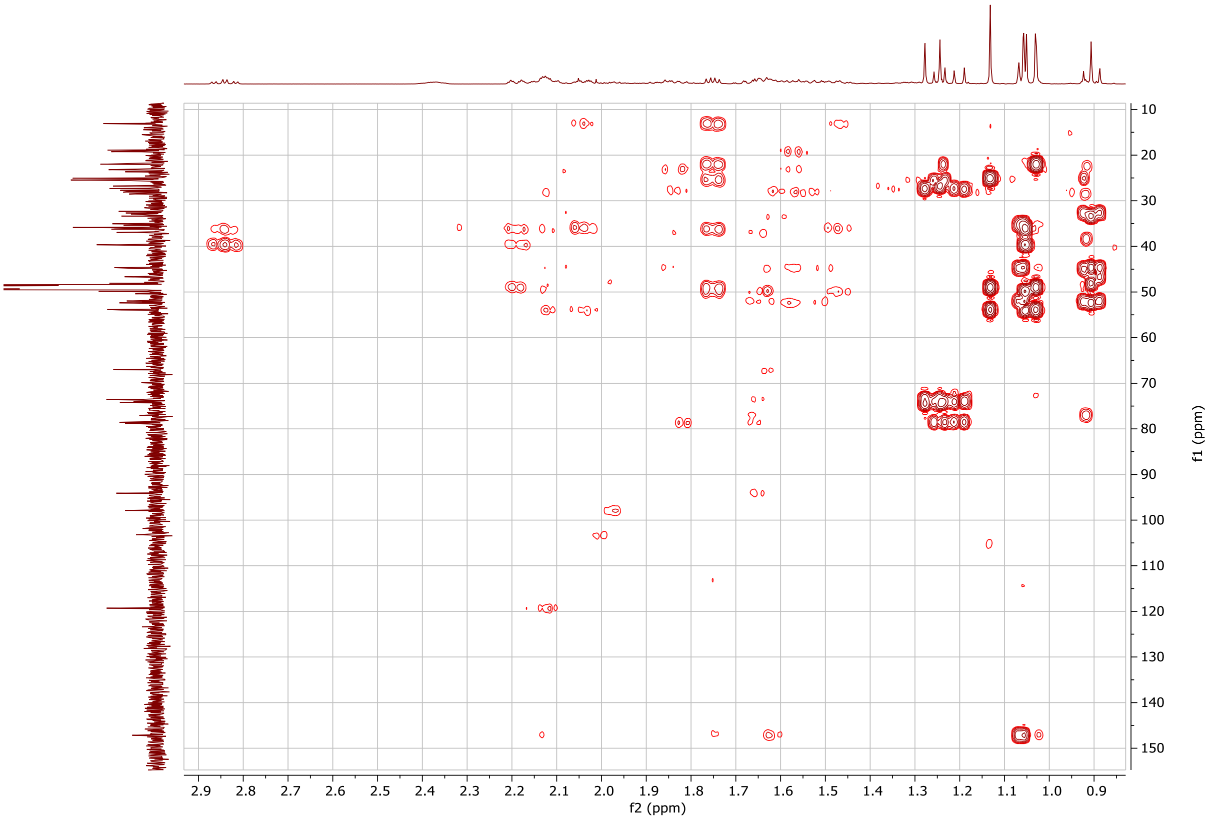

Figure S8a. HMBC (CD_3OD) expanded spectrum of compound **1abc**

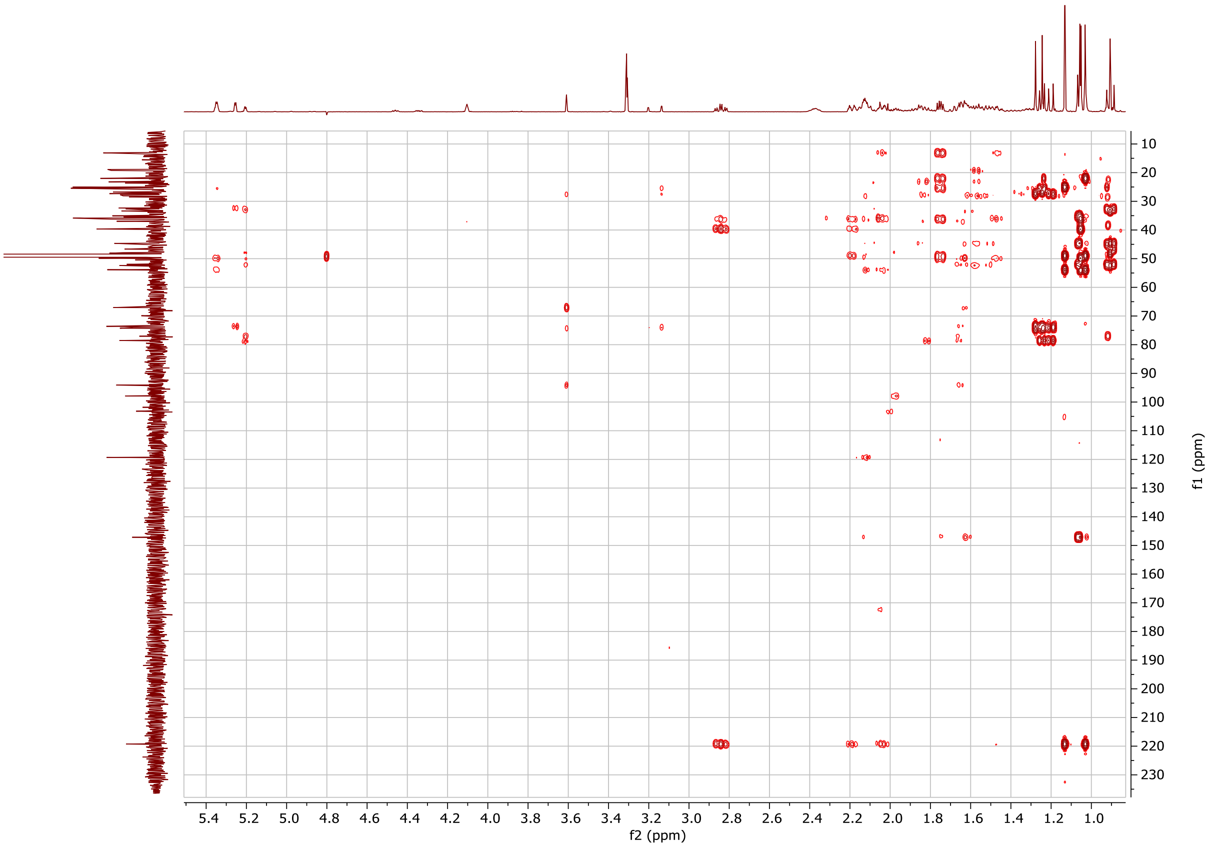

Figure S8b. HMBC (CD_3OD) expanded spectrum of compound **1abc**

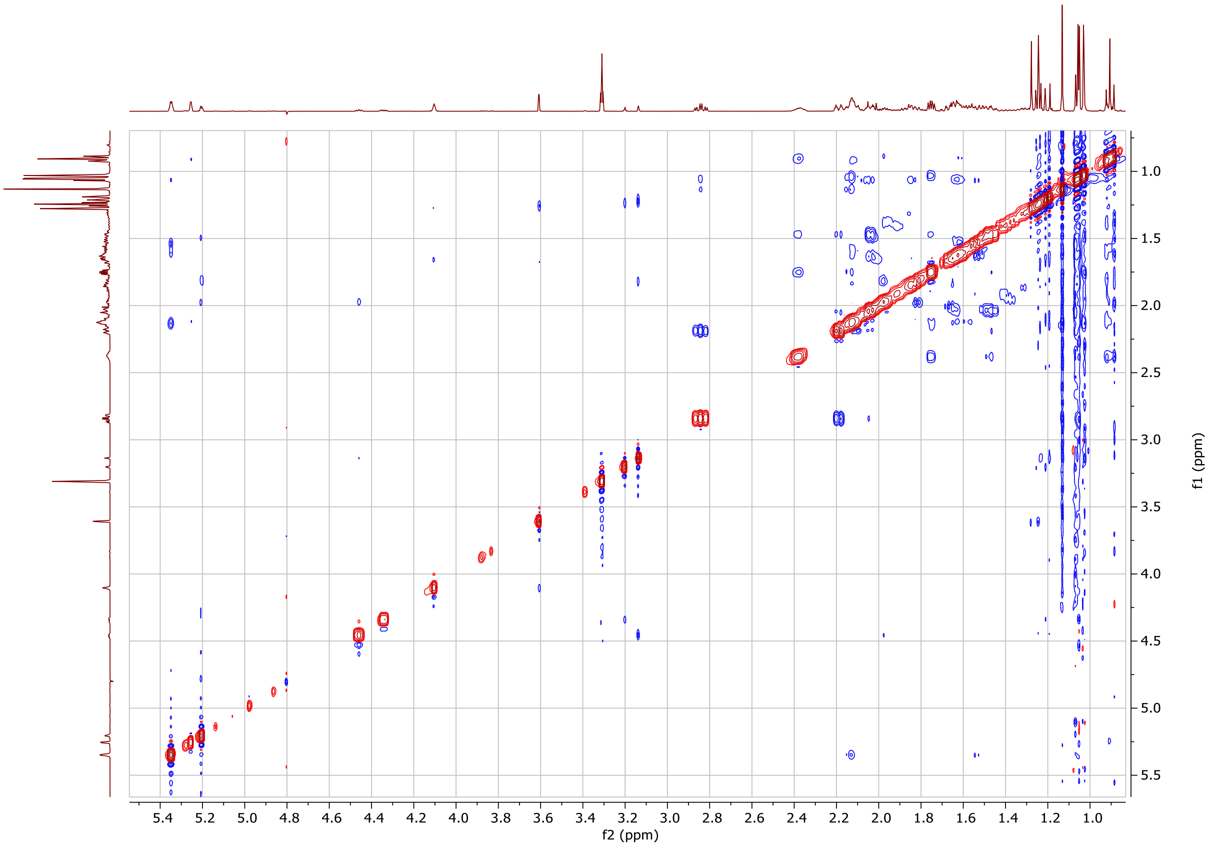

Figure S9. NOESY (CD_3OD) full spectrum of compound **1abc**

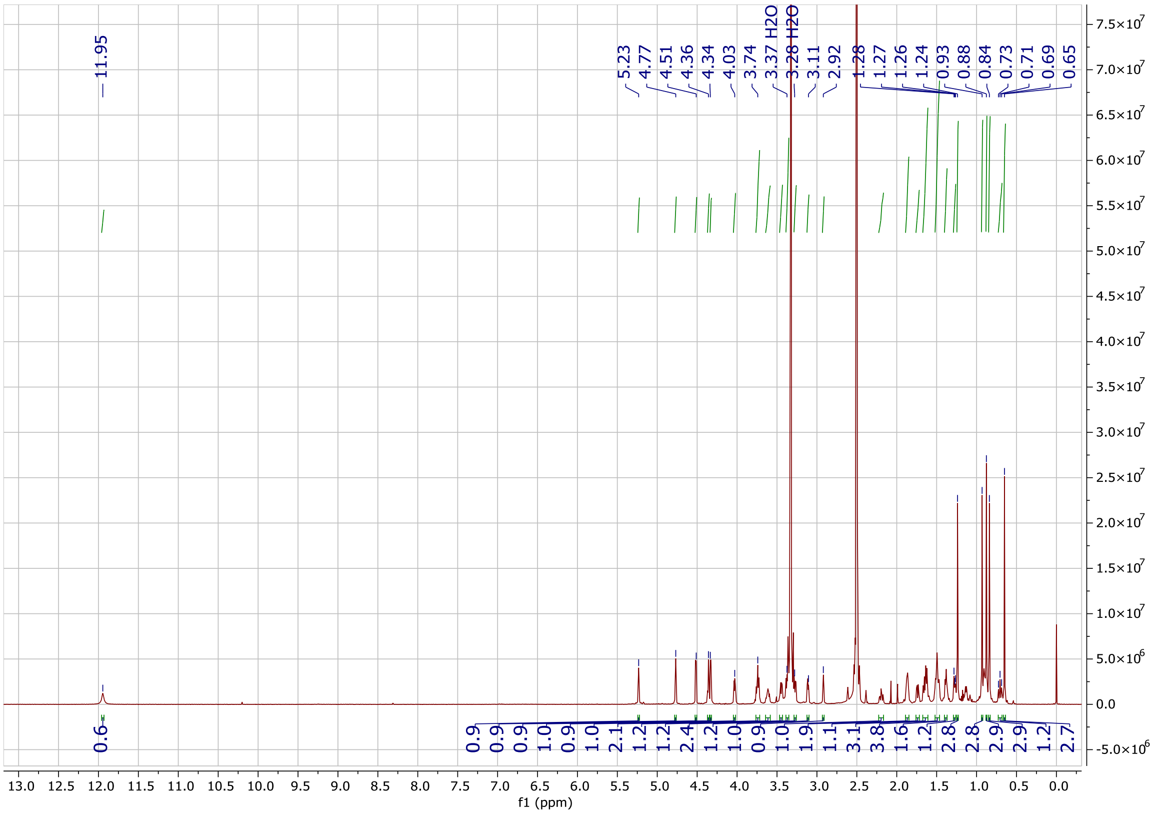

Figure S10. ^{1}H-NMR (600 MHz, DMSO-*d*6) spectrum of compound **2**

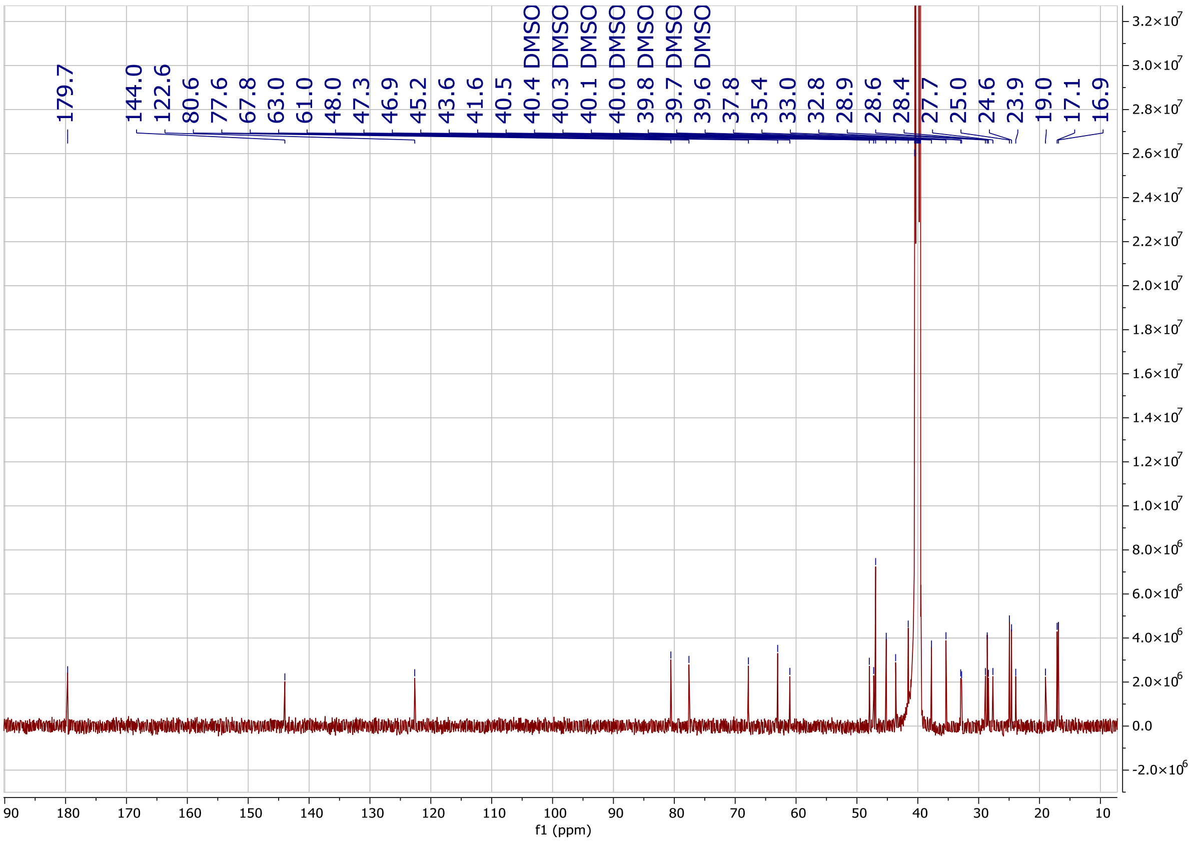

Figure S11. ^{13}C-NMR (150 MHz, DMSO-d_6) spectrum of compound **2**

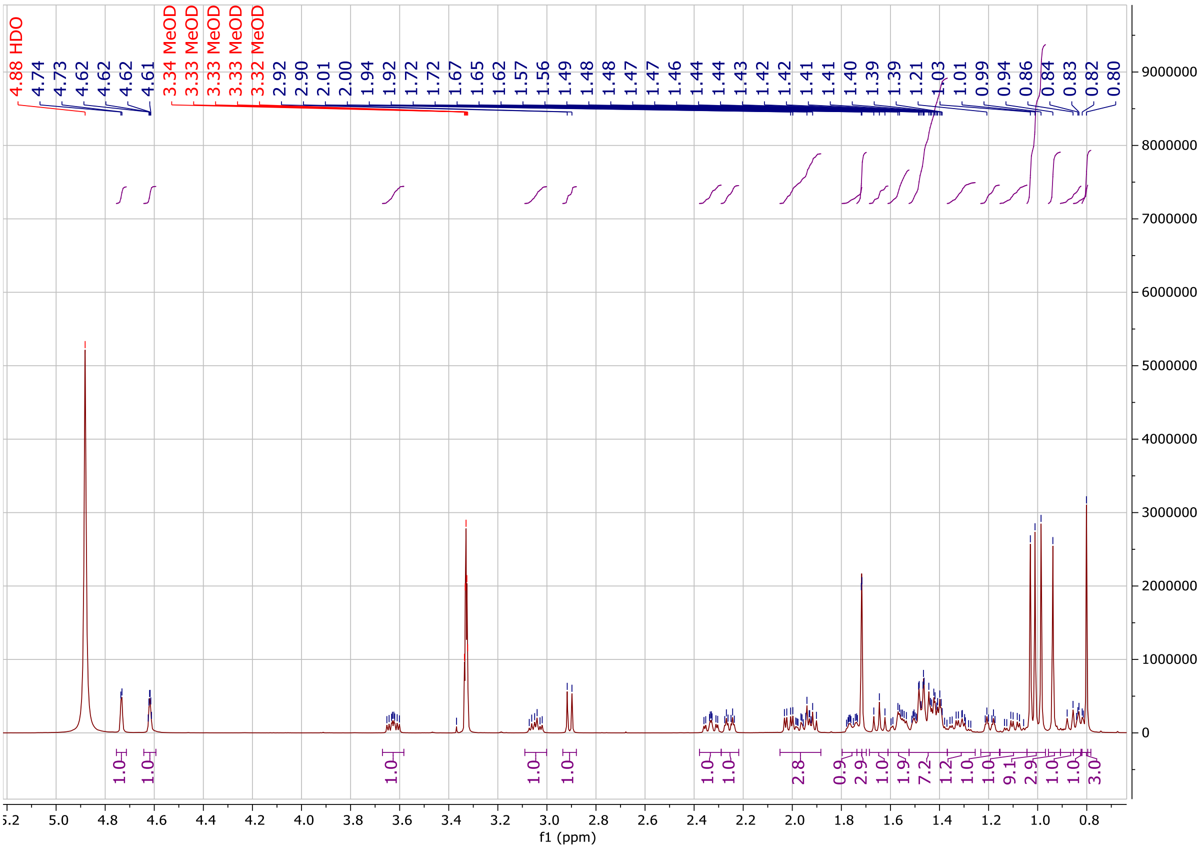

Figure S12. ^{1}H-NMR **(**500 MHz, CD_3OD**)** spectrum of compound **3**

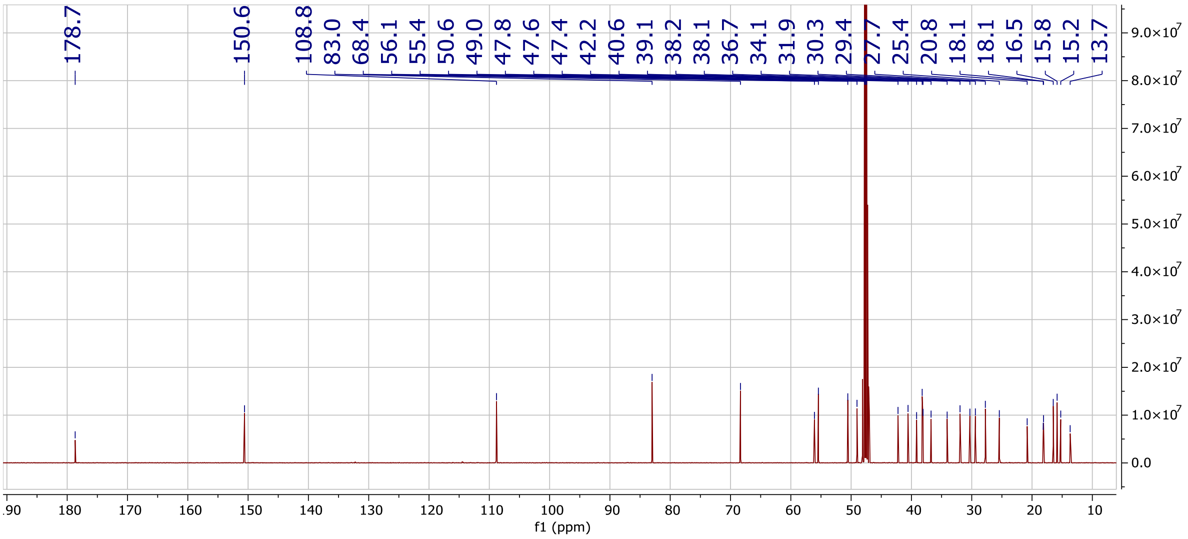

Figure S13. ^{13}C-NMR (125 MHz, CD_3OD) spectrum of compound **3**

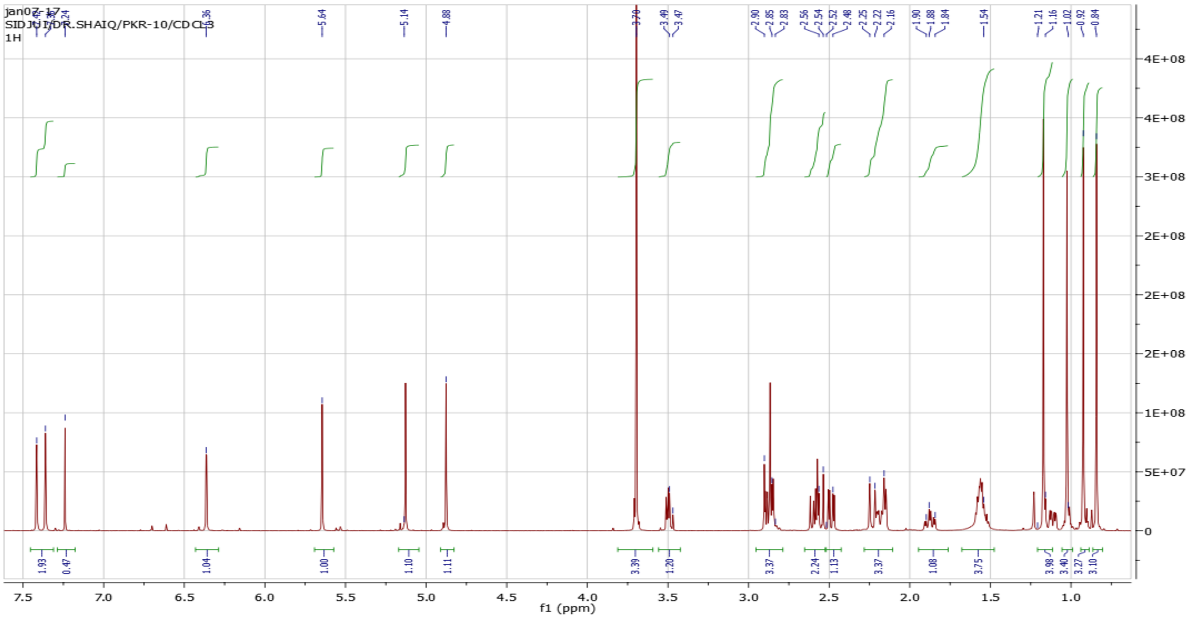

Figure S14. ^{1}H-NMR **(**500 MHz, $CDCl_3$**)** spectrum of compound **4**

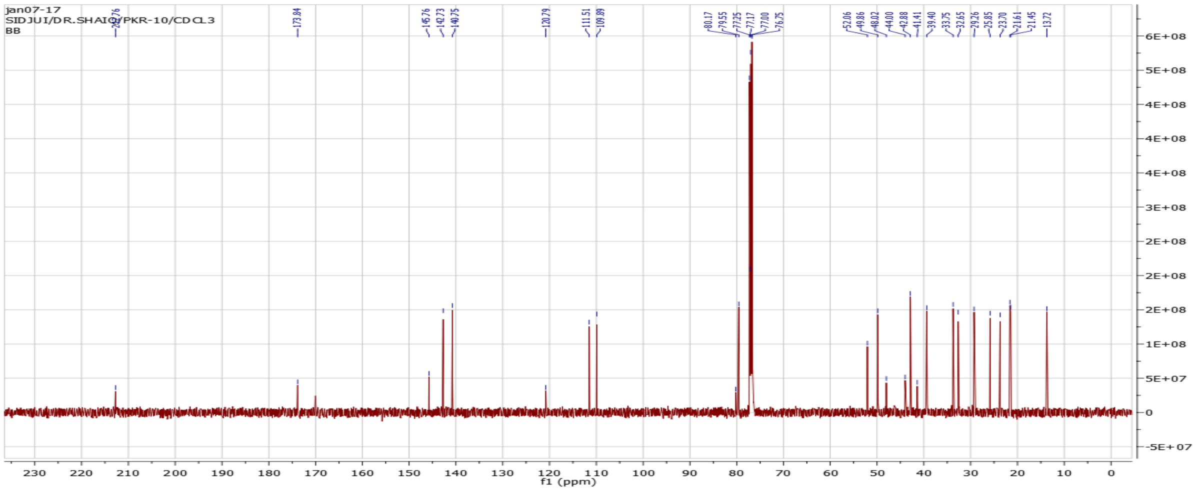

Figure S15. ^{13}C-NMR (125 MHz, $CDCl_3$) spectrum of compound **4**

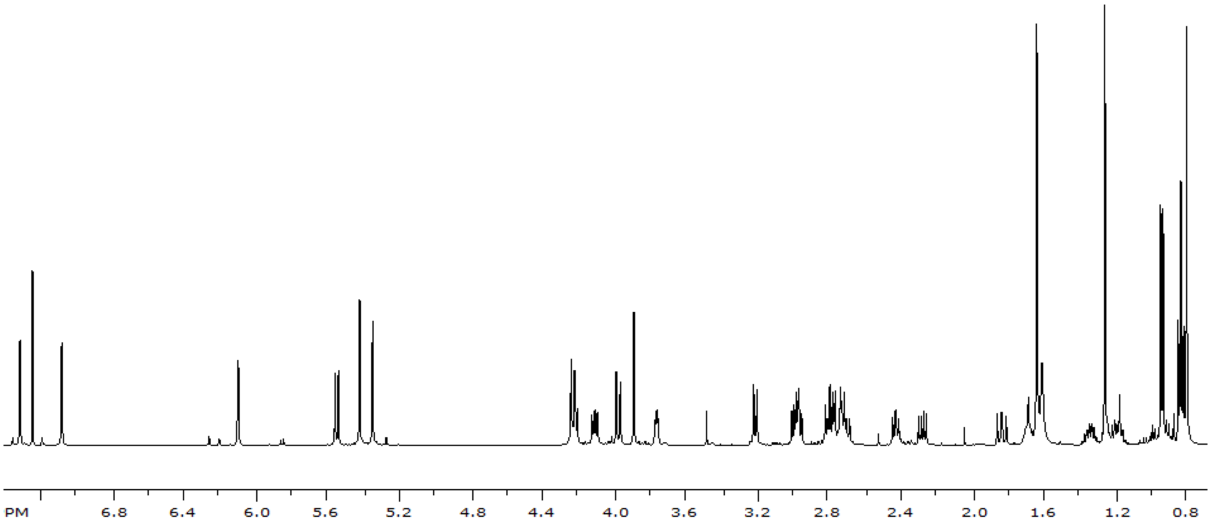

Figure S16. ^{1}H-NMR (500 MHz, $CDCl_3$) spectrum of compound **5**

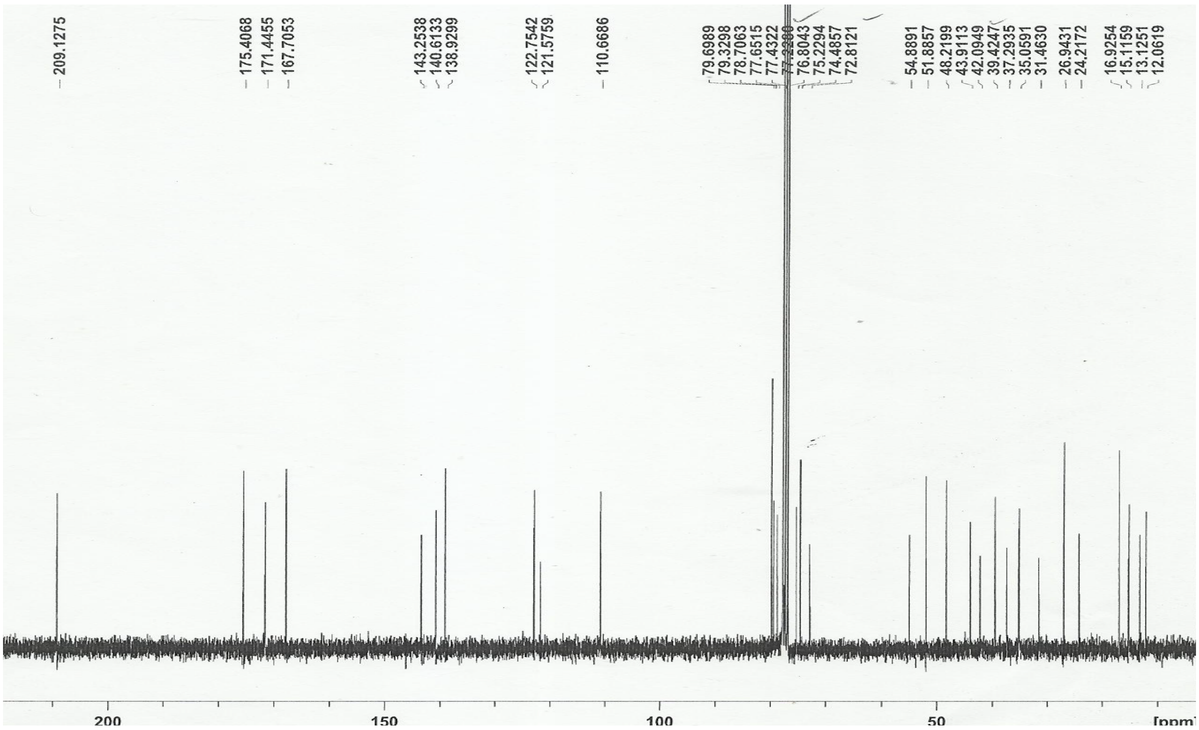

Figure S17. ^{13}C-NMR (125 MHz, $CDCl_3$) spectrum of compound **5**

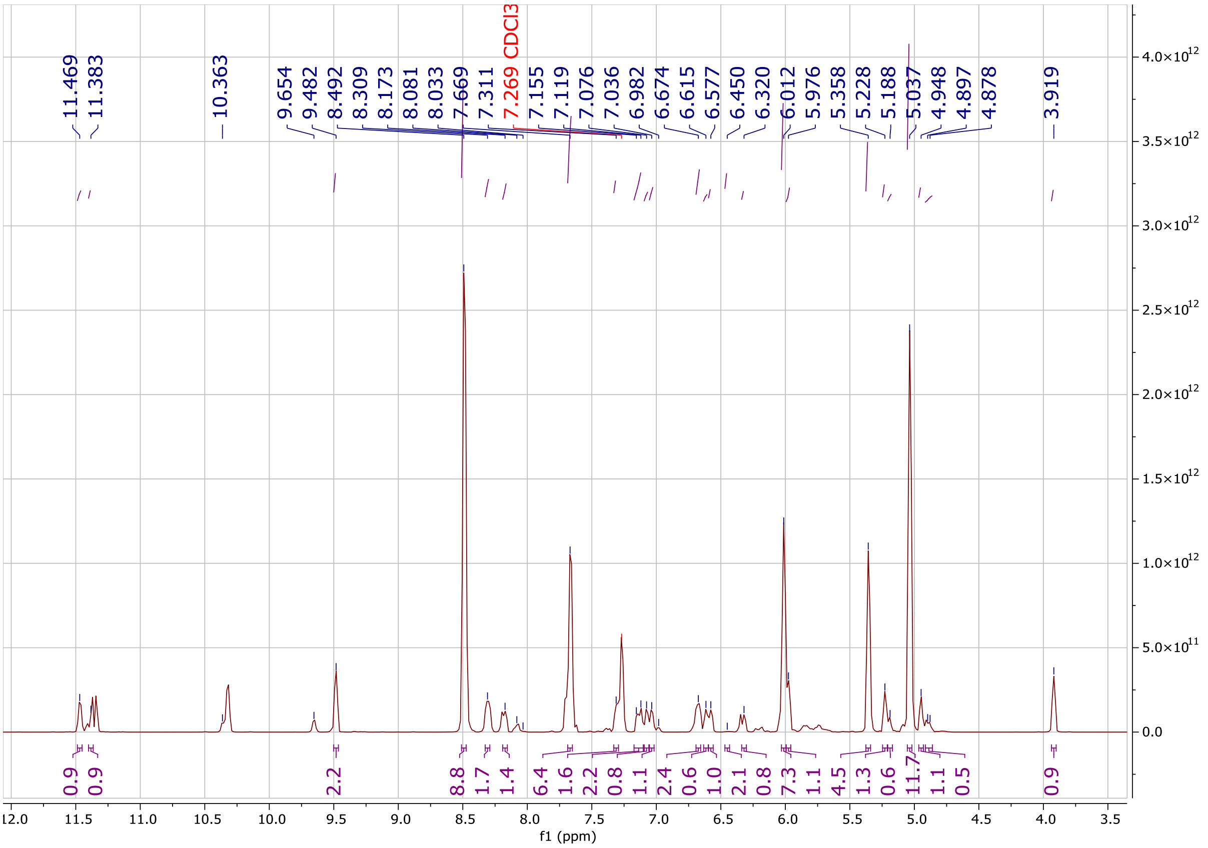

Figure S18. ^{1}H-NMR (500 MHz, $CDCl_3$) spectrum of compound **6**

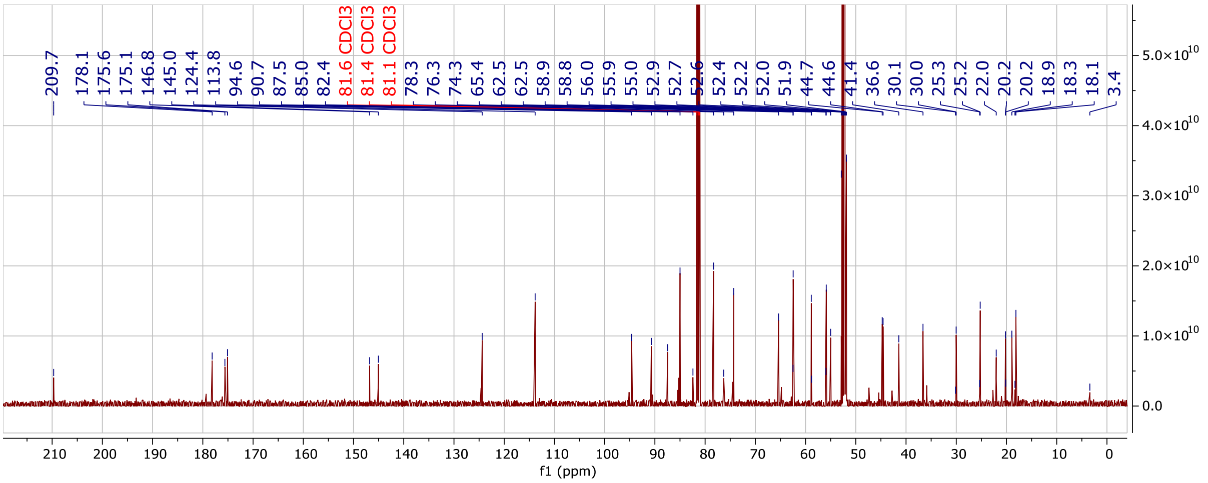

Figure S19. ^{13}C-NMR (125 MHz, $CDCl_3$) spectrum of compound **6**

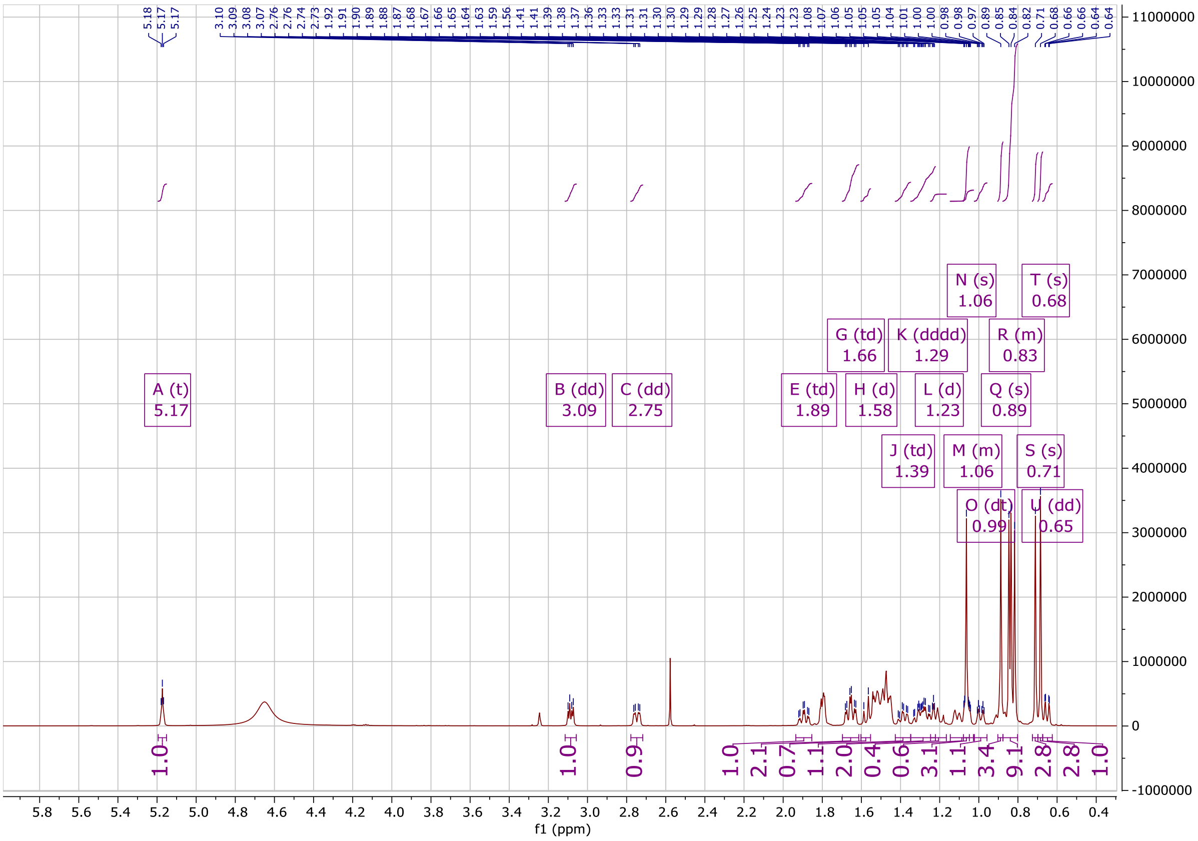

Figure S20. ^{1}H-NMR (600 MHz, CD_3OD) spectrum of compound **7**

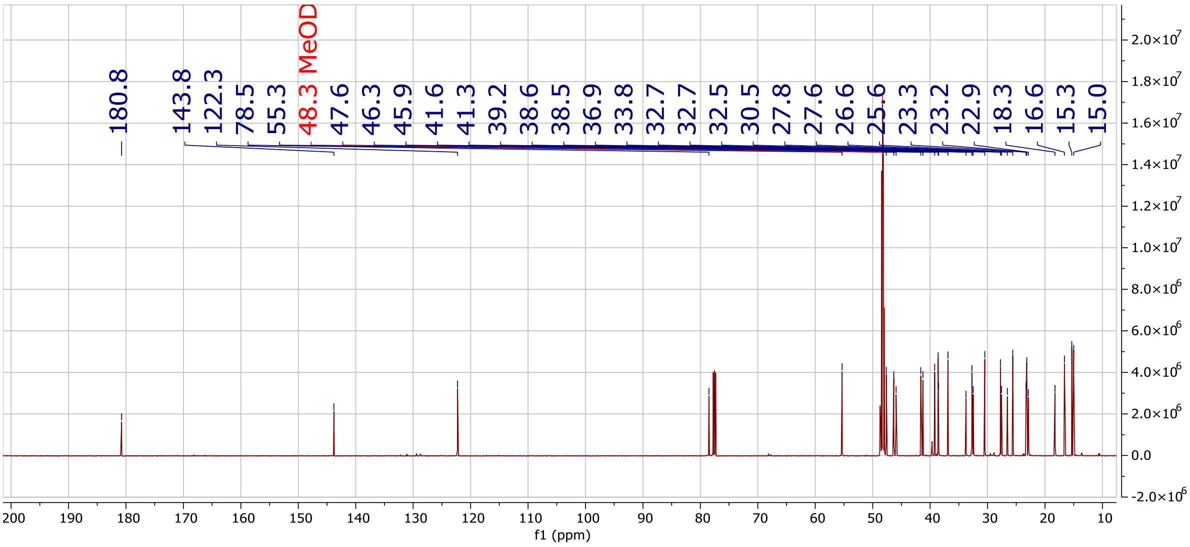

Figure S21. ^{13}C-NMR (150 MHz, CD_3OD) spectrum of compound **7**

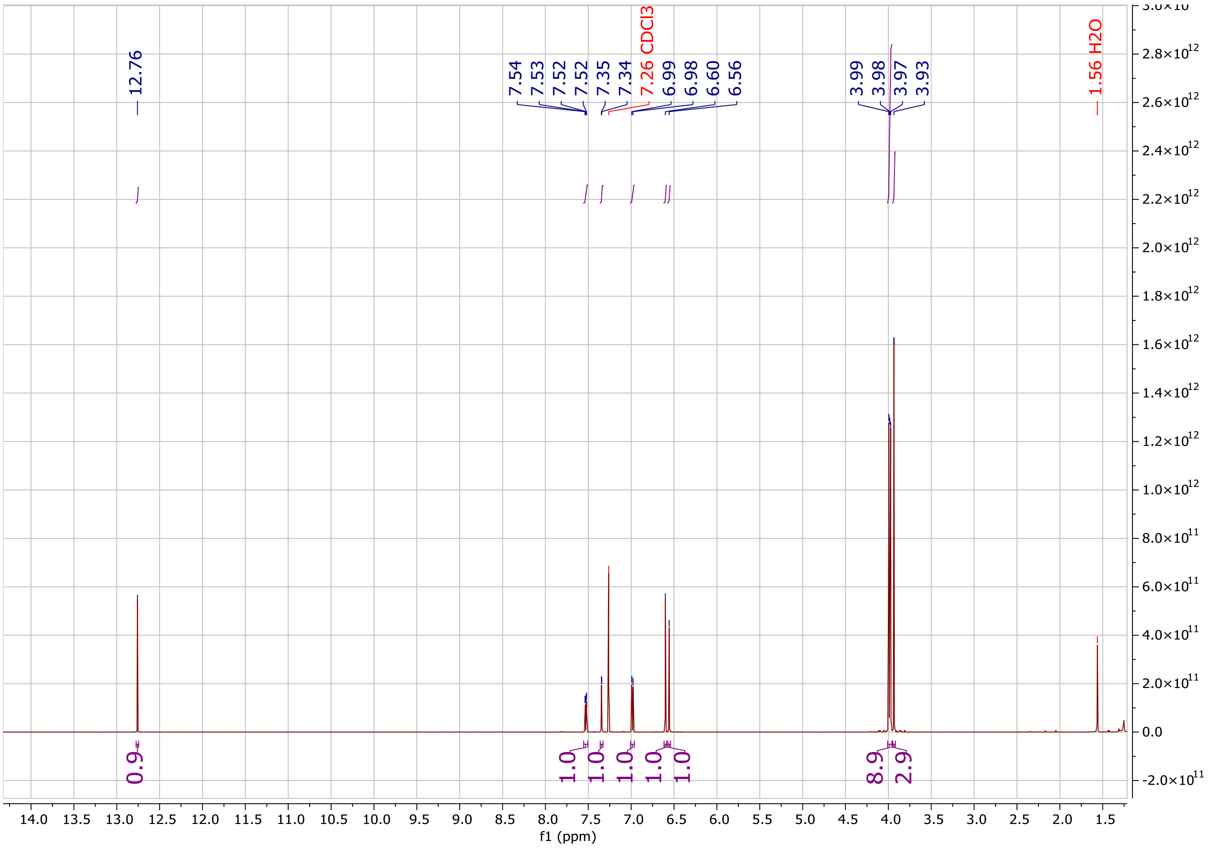

Figure S22. ^{1}H-NMR **(**600 MHz, $CDCl_3$**)** spectrum of compound **8**

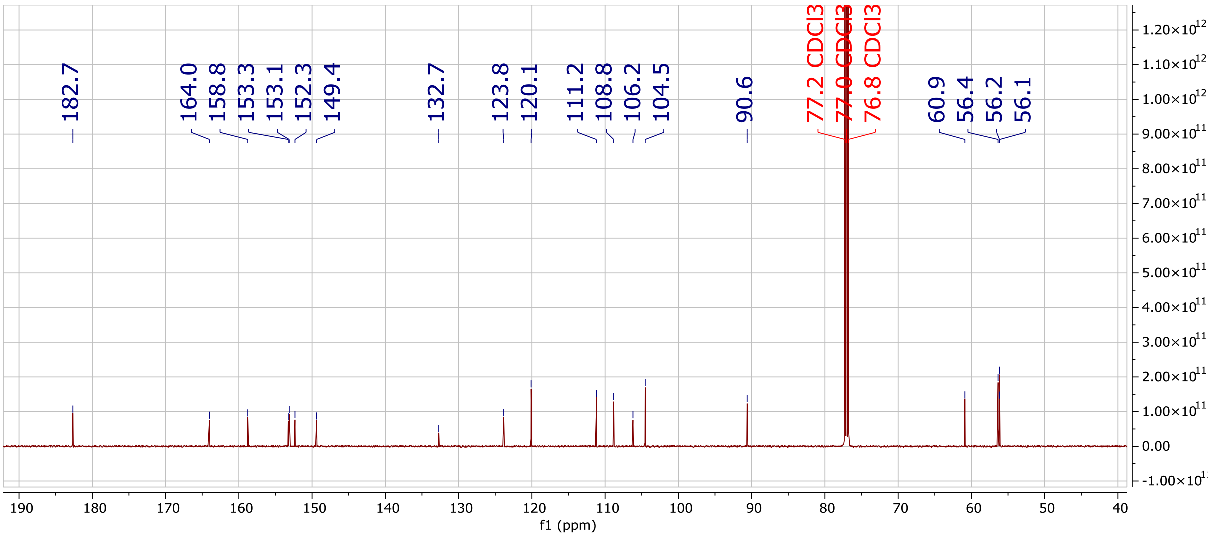

Figure S23. ^{13}C-NMR (150 MHz, $CDCl_3$) spectrum of compound **8**

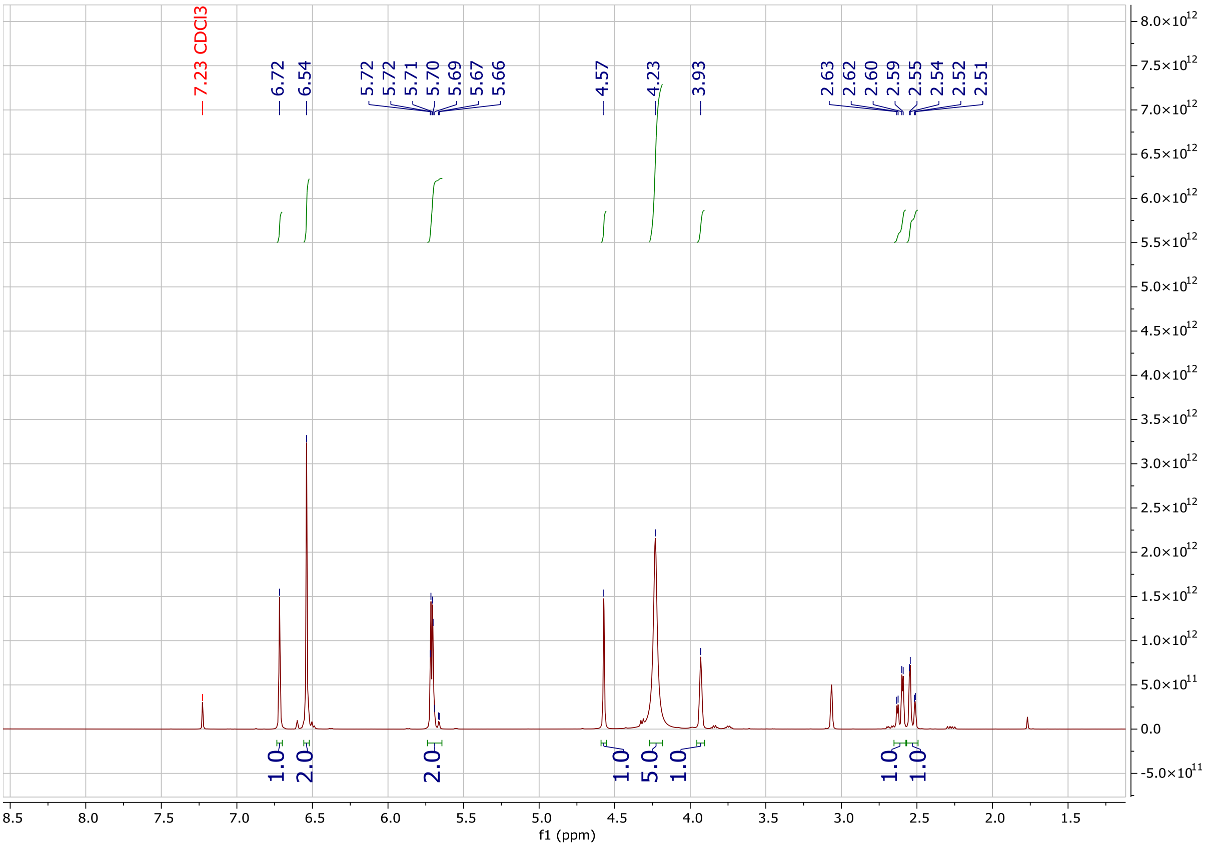

Figure S24. ^{1}H-NMR (600 MHz, $CDCl_3$) spectrum of compound **9**

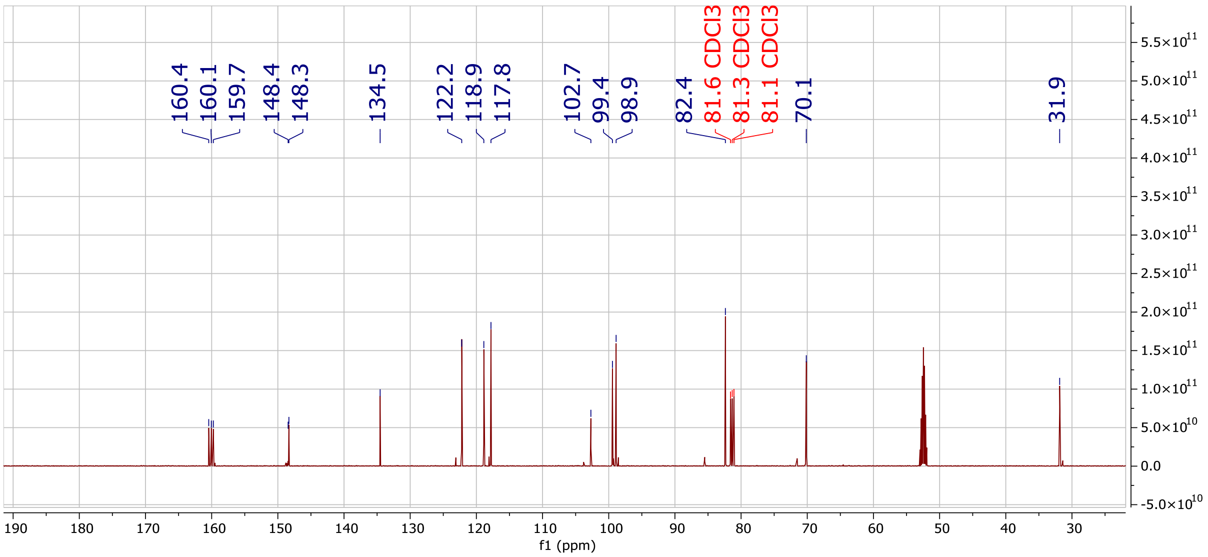

Figure S25. ^{13}C-NMR (150 MHz, $CDCl_3$) spectrum of compound **9**

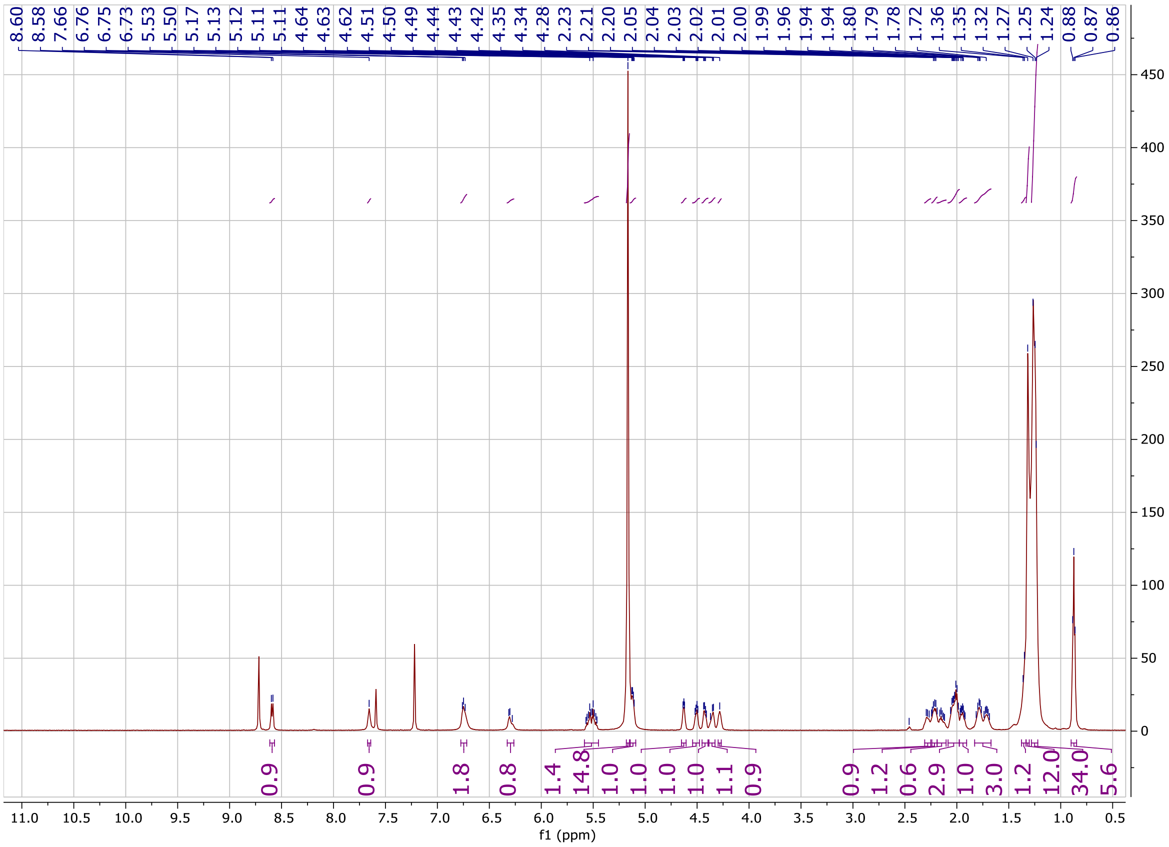

Figure S26. ^{1}H-NMR **(**600 MHz, Pyridine-d_5**)** spectrum of compound **10**

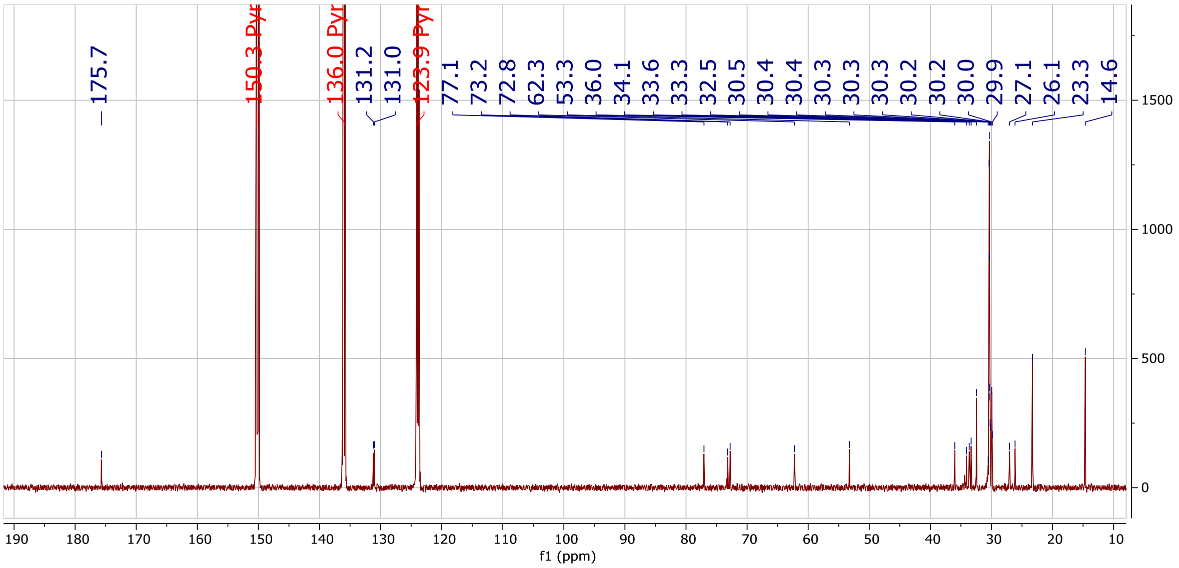

Figure S27. ^{13}C-NMR (150 MHz, Pyridine-*d*5) spectrum of compound **10**

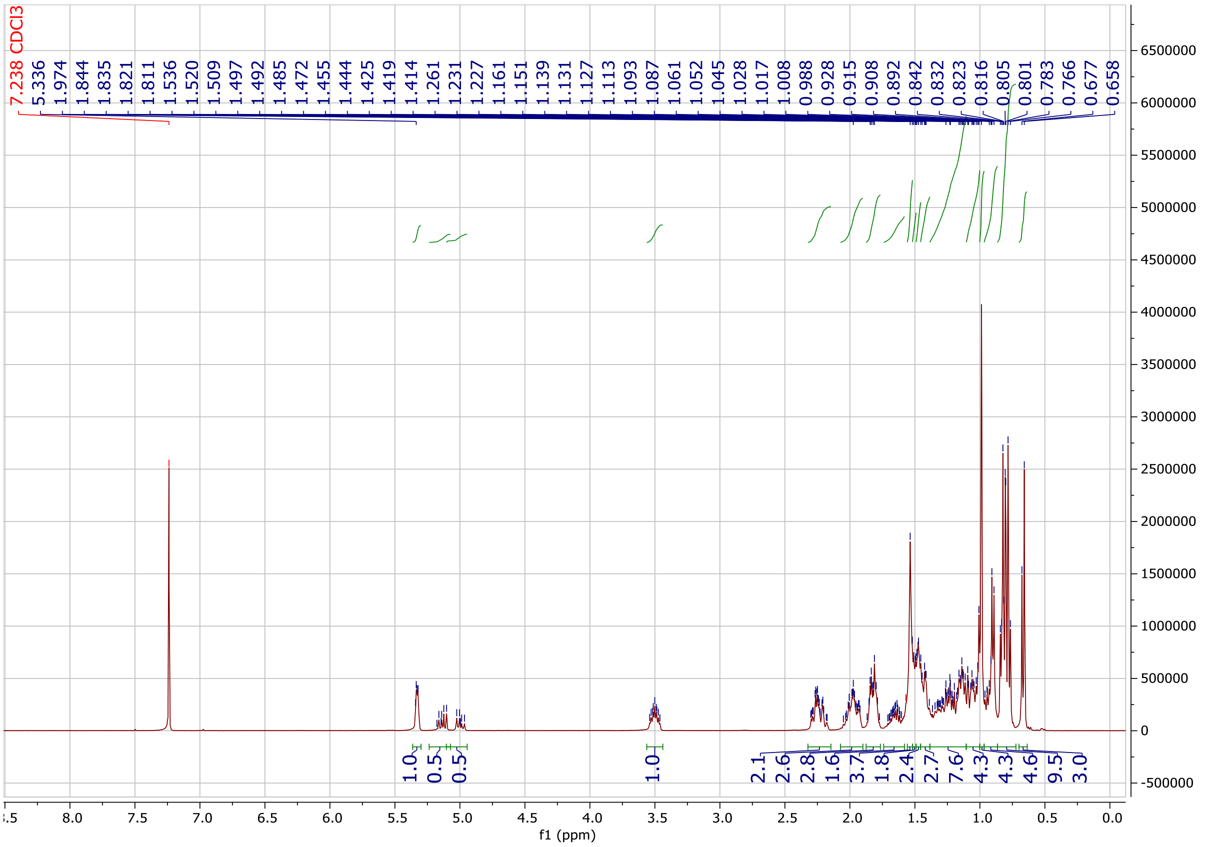

Figure S28. ^{1}H-NMR (CDCl3, 500 MHz) spectrum of the mixture of compounds **11** and **13**

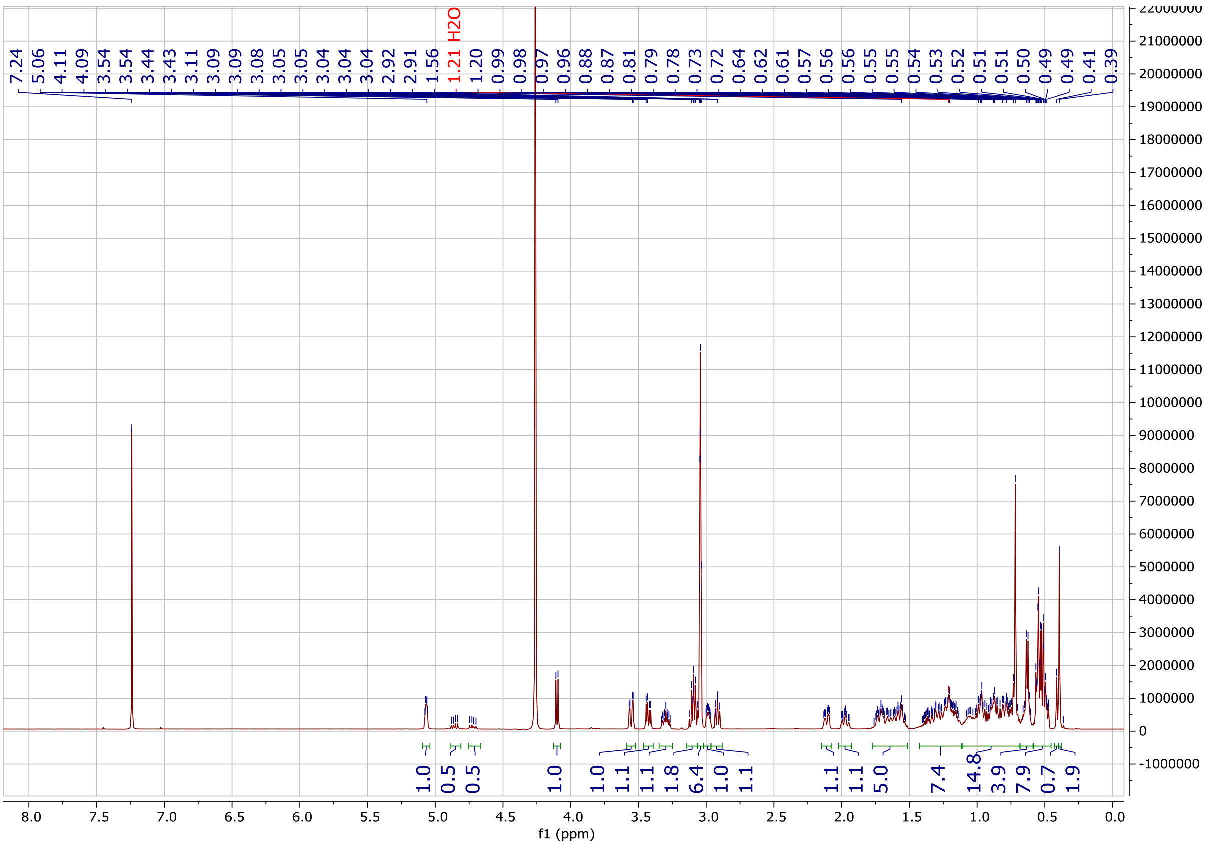

Figure S29: ^{1}H-NMR ($CDCl_3$, 500 MHz) spectrum of the mixture of compounds **12** and **14**
